# Hydrogen, Bicarbonate, and Their Associated Exchangers in Cell Volume Regulation

**DOI:** 10.3389/fcell.2021.683686

**Published:** 2021-06-24

**Authors:** Yizeng Li, Xiaohan Zhou, Sean X. Sun

**Affiliations:** ^1^Department of Mechanical Engineering, Kennesaw State University, Marietta, GA, United States; ^2^Department of Physics, University of Toronto, Toronto, ON, Canada; ^3^Department of Mechanical Engineering, Johns Hopkins University, Baltimore, MD, United States; ^4^Institute for NanoBioTechnology, Johns Hopkins University, Baltimore, MD, United States; ^5^Center for Cell Dynamics, Johns Hopkins School of Medicine, Baltimore, MD, United States

**Keywords:** cell volume regulation, pH, hydrogen, bicarbonate, sodium-hydrogen exchanger, chloride-bicarbonate exchanger, sodium-bicarbonate cotransporter, sodium-potassium exchanger

## Abstract

Cells lacking a stiff cell wall, e.g., mammalian cells, must actively regulate their volume to maintain proper cell function. On the time scale that protein production is negligible, water flow in and out of the cell determines the cell volume variation. Water flux follows hydraulic and osmotic gradients; the latter is generated by various ion channels, transporters, and pumps in the cell membrane. Compared to the widely studied roles of sodium, potassium, and chloride in cell volume regulation, the effects of proton and bicarbonate are less understood. In this work, we use mathematical models to analyze how proton and bicarbonate, combined with sodium, potassium, chloride, and buffer species, regulate cell volume upon inhibition of ion channels, transporters, and pumps. The model includes several common, widely expressed ion transporters and focuses on obtaining generic outcomes. Results show that the intracellular osmolarity remains almost constant before and after cell volume change. The steady-state cell volume does not depend on water permeability. In addition, to ensure the stability of cell volume and ion concentrations, cells need to develop redundant mechanisms to maintain homeostasis, i.e., multiple ion channels or transporters are involved in the flux of the same ion species. These results provide insights for molecular mechanisms of cell volume regulation with additional implications for water-driven cell migration.

## 1. Introduction

Mammalian cell volume regulation is an essential process during the cell life cycle (Lang et al., 1998; Kay, 2017; Perez-Gonzalez et al., 2019; Li et al., 2020). Seventy percent of the cell volume fraction consists of incompressible water. Since large molecules are not permeable through the cell membrane, on short time scales where protein production is negligible, water flux in and out of the cell is the primary process that determines the cell volume variation. Water flux across the cell membrane follows hydraulic pressure and osmotic pressure differences (Lang et al., 1998; Tao et al., 2017; Li et al., 2020). The intracellular osmotic pressure arises from the entropy of mixing of water with large molecules that are non-permeable through the membrane and small permeable solutes such as ions and sugar. Major ionic species in cells include Na^+^, K^+^, Cl^−^, ${\text{HCO}}_{3}^{-}$, Ca^2+^, and H^+^. The cell membrane embeds many different types of channels, transporters, and pumps that allow ions to move in and out of the cell. A net ion flux may lead to a net change in the intracellular osmolarity, which drives water flux, cell volume change, and cell boundary movement (Li et al., 2020).

Unlike Na^+^, K^+^, and Cl^−^ which are non-reactive, ${\text{HCO}}_{3}^{-}$ and H^+^ are reactive, and their concentrations are correlated. The intracellular concentration of a reactive species is not solely determined by its flux across the cell membrane. Transporters such as the Na^+^/H^+^ and Cl^−^/${\text{HCO}}_{3}^{-}$ exchangers generate ion fluxes for both non-reactive and reactive species. Inhibition of these transporters can potentially lead to chemical reactions in the cell, which modulate the cell volume in a non-trivial manner. With emerging data showing the importance of ion and water fluxes during cell migration, force generation, and morphological change (Klein et al., 2000; Loitto et al., 2002; Stock et al., 2005; Lauritzen et al., 2012; Stroka et al., 2014; Li and Sun, 2018; Li et al., 2019, 2020), it becomes essential to understand the regulation of intracellular ${\text{HCO}}_{3}^{-}$ and H^+^. In this work, we investigate how ${\text{HCO}}_{3}^{-}$ and H^+^ are involved in cell volume regulation when the activities of relevant ion transporters are perturbed. We will further discuss counter-intuitive ionic concentration changes during ion channel/transporter/pump perturbation.

In general, ions are transported across membranes both passively and actively. Passive ionic transport is carried out by ion-specific channels and pores, some of which are sensitive to cortical tension. Active transport is carried out by energy-consuming ion pumps, which utilize chemical energy (ATP) to transport ions against a chemical potential gradient (Gadsby, 2009). Another type of active transporters is classified as secondary active transporters, which utilize the chemical-electrical potential gradient of one species to facilitate the transport of another species (Shi, 2013). Given the molecular complexity in cell ionic homeostasis, various ion channels/transporters/pumps and the associated fluxes must be considered together. Realistic computational models are necessary to make significant progress. Here we will use mathematical analysis to predict the change of ion concentration.

**Figure 1:**
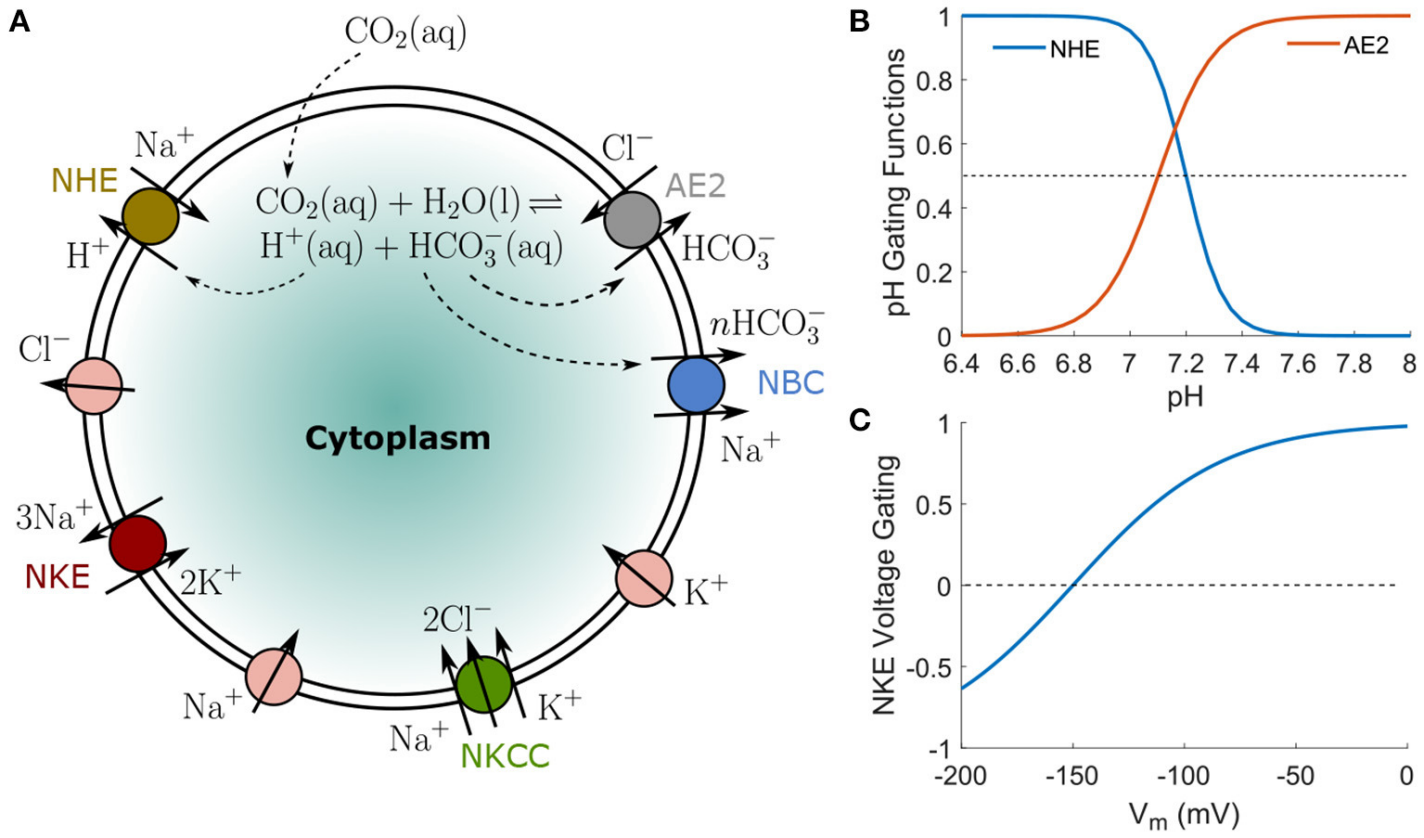
**(A)** A schematic representation of ion pumps, transporters and passive channels included in the model to compute cell ion homeostasis. The ion fluxes can depend on intracellular and extracellular ion concentrations, transmembrane potential, the tension in the cell cortex, and expression/activation levels of each channel (see Methods). The overall cell ionic composition depends on ion channel/transporter/pump expression and activation. **(B)** pH-gating profiles of NHE and AE2 transporters, taken from data in Casey et al. (2010). **(C)** Voltage-gating profile for NKE, taken from data in Gadsby et al. (1985).

In this paper, we include the following transporters: Na^+^/K^+^ exchanger (NKE), Na^+^-K^+^-Cl^−^ cotransporter (NKCC), Na^+^/H^+^ exchanger (NHE), Cl^−^/${\text{HCO}}_{3}^{-}$ exchanger (AE2), and Na^+^/${\text{HCO}}_{3}^{-}$ cotransporter (NBC) (Figure 1A). The NKE is a ubiquitous and important active ion pump that maintains the membrane potential of cells. It exports three Na^+^ ions and intakes two K^+^ ions per ATP molecule. Because the overall flux is positive outwards, the pump's activity depends on the membrane potential (Gadsby et al., 1985). The NKE flux also depends on the concentrations of Na^+^ and K^+^ (Gao et al., 1995; Bueno-Orovio et al., 2014) and saturates at high concentration limits (Bueno-Orovio et al., 2014). The Na^+^-K^+^-Cl^−^ cotransporter (NKCC), along with its isoforms, is widely expressed in various cell types (Russell, 2000). The NKCC simultaneously transports one Na^+^, one K^+^, and two Cl^−^s into the cell under physiological conditions. The Na^+^/H^+^ exchanger (NHE), which has ten identified isoforms, is expressed in almost all tissues (Vallés et al., 2015). It imports one Na^+^ and extrudes one H^+^ under physiological conditions. This exchanger has significant effects on water flux, cell volume regulation (Cala, 1980; Ericson and Spring, 1982; Grinstein et al., 1986; Alexander and Grinstein, 2006; Maeno et al., 2006; Hughes et al., 2010), cell migration (Scholz et al., 1995; Lauritzen et al., 2012; Chang et al., 2014; Stroka et al., 2014), and is a major therapeutic target (Mahnensmith and Aronson, 1985; Karmazyn, 2013; Vallés et al., 2015). NHE is quiescent at intracellular pH > 7.2 (Casey et al., 2010). The Cl^−^/${\text{HCO}}_{3}^{-}$ exchanger (AE2), which imports one Cl^−^ and extrudes one ${\text{HCO}}_{3}^{-}$, is also common in cells. This exchanger is almost quiescent at intracellular pH < 6.8 − 7.3. The Na^+^/${\text{HCO}}_{3}^{-}$ cotransporter (NBC), which transports Na^+^ and ${\text{HCO}}_{3}^{-}$ in the same direction, is expressed in both epithelial and non-epithelial cells (Boron and Boulpaep, 1989). The stoichiometry of this cotransporter is cell-type dependent (Gross et al., 2001) and is either ${\text{HCO}}_{3}^{-}:{\text{Na}}^{+}=3:1$ or 2:1 (Boron and Boulpaep, 1989; Weinstein, 2013). As a result, NBC is electrogenic and influences the intracellular pH (Boron and Boulpaep, 1989). Depending on the concentrations of the two species and the membrane potential, the flux of NBC can be either in or out of the cell.

Given the large amount of identified ion transporters so far, the ones we include in the model are not meant to be exhaustive, nor do we intend to model the exact expression and activity levels of channels, transporters, and pumps in various cell types (Hoffman, 1969; Landowne and Ritchie, 1970; Baker and Willis, 1972; Burnham and Stirling, 1984a,b; Russell, 2000; Bonar and Casey, 2008; Blaesse et al., 2009; Romero et al., 2013; Vallés et al., 2015), although by varying flux parameters in the model, we can computationally examine cell responses when the activity levels of ion channels, transporters, and pumps are varied. In this work, we will focus on the generic interaction among hydrogen, bicarbonate, and their associated exchangers. A possible explanation of NHE-dependent regulatory cell volume increases will be discussed. Moreover, we demonstrate explicitly how ionic fluxes and cytoskeletal contraction can work together to achieve the final observed cell volume. We also examine how ion transporter expression in several common cell types can lead to variations in cell properties such as cell volume, pH, and transmembrane voltage and compare with experimental results.

## 2. Materials and Methods

### 2.1. Model Description

We present a cell volume regulation model through the influence of ion fluxes, intracellular osmolarity, and pH on the time scale of minutes. In the model, we consider the following intracellular species, Na^+^, K^+^, Cl^−^, H^+^, ${\text{HCO}}_{3}^{-}$, A^−^, Buf^−^, and HBuf, where A^−^ represents charged organic molecules or proteins that are not permeable across the cell membrane. The total number of intracellular proteins *N*_*A*_ is prescribed. The organic molecules or proteins have various charges, and the average charge is negative (Gitlin et al., 2006). We thus lump all the proteins and assume an effective average valence of −1. The impact of the average valence will be discussed. Within the cell, we include an unprotonated buffer (Buf^−^) and protonated buffer (HBuf) species, both of which are considered as non-permeable across the cell membrane (Weinstein, 1986) and the total, *N*_Buf_ + *N*_HBuf_, is fixed. The extracellular ion concentrations $c_{n}^{0}$ are given based on generic cell culture medium composition or extracellular fluid environment *in vivo*. $c_{A}^{0}$ is adjusted based on the concentration of other charged ion species so that the electro-neutrality condition of the medium is satisfied. Here *c* represents concentrations, and the superscript ‘0’ indicates quantities associated with the extracellular environment. We can also include large, non-permeable molecules (G) in the extracellular space to adjust the baseline osmolarity of the medium, in a similar manner as modulating the extracellular hydrostatic pressure, which is determined up to a constant. By default, we let this molecule to be charge-free, but it can assume any valence to ensure the electroneutrality of the extracellular space when other extracellular species are specified. Since the molecule is non-permeable and the electroneutrality condition in the extracellular domain is maintained, it will not affect the membrane potential nor voltage-dependent ion channels, transporters, and pumps.

Considering a generic spherical cell experiencing volume regulation, the rate change of the cell radius, *r*, is modulated by osmotic and hydrostatic pressure gradients (Li et al., 2020).

(1) $$\begin{array}{l} \frac{dr}{dt}=J_{\text{water}}=-\alpha_{\text{w}}\left(\Delta p-\Delta \Pi \right), \end{array}$$

where *J*_water_ is water flux across the cell membrane, defined positive inward. α_w_ is the combined permeability coefficient of water from the lipid membrane and aquaporins (AQPs). The water permeability through lipids is several orders of magnitude smaller than that of AQPs. So in most cases α_w_ is dominated by the contribution from AQPs. $\Delta \Pi =RT\left(\underset{n}{\sum }c_{n}-\underset{n}{\sum }c_{n}^{0}\right)$ is the osmotic pressure difference across the cell membrane. The extracellular and intracellular osmolytes in the model are, respectively, $c_{n}={\left\{c_{\text{Na}},c_{\text{K}},c_{\text{Cl}},c_{\text{H}},c_{\text{HC}{\text{O}}_{3}},c_{\text{A}},c_{\text{Buf}},c_{\text{HBuf}}\right\}}^{T}$ and $c_{n}^{0}={\left\{c_{\text{Na}}^{0},c_{\text{K}}^{0},c_{\text{Cl}}^{0},c_{\text{H}}^{0},c_{\text{HC}{\text{O}}_{3}}^{0},c_{\text{A}}^{0},c_{\text{G}}^{0}\right\}}^{T}$. The hydrostatic pressure difference across the cell membrane, Δ*p*, is related to the combined effective cortical and membrane stress, σ, by the Laplace law Δ*p* = 2*hσ*/*r*, where *h* is the combined effective thickness of the cell cortex and membrane. The stress can be obtained from the constitutive relation of the actomyosin cortex and membrane (Jiang and Sun, 2013) and is composed of two parts, σ = σ_*p*_+σ_*a*_, where σ_*p*_ is the passive mechanical stress of the actin network and membrane, which includes contributions from F-actin crosslinkers, filament mechanics, and lipid bilayer. σ_*a*_ is the active myosin contraction from the cortex. The passive stress is typically small compared to the active stress, and in this model, we only consider the active stress such that σ ≃ σ_*a*_. Hereafter, we will use cortical stress to refer to the combined stress from the cortical layer and the cell membrane. Similarly, we will use cortical tension and cortical thickness to refer to the combined effects from both the cortical layer and the lipid bilayer.

The chemical equilibrium equation for the bicarbonate-carbonic acid pair is ${\text{CO}}_{2}\left(\text{aq}\right)+{\text{H}}_{2}\text{O}\left(\text{l}\right)⇌{\text{H}}^{+}\left(\text{aq}\right)+{\text{HCO}}_{3}^{-}\left(\text{aq}\right)$, where [CO_2_]_aq_ is related to the partial pressure of CO_2_, *P*_CO_2__, by the Henry constant *k*_*H*_, i.e., [CO_2_]_aq_ = *P*_CO_2__/*k*_*H*_. The reaction equilibrium constant for the bicarbonate-carbonic acid pair is $k_{c}={\left[{\text{HCO}}_{3}^{-}\right]}_{\text{aq}}{\left[{\text{H}}^{+}\right]}_{\text{aq}}/{\left[{\text{CO}}_{2}\right]}_{\text{aq}}$. Defining ${\text{pH}}_{0}=-{\text{log}}_{10}{\left[{\text{H}}^{+}\right]}_{\text{aq},0}$ as the extracelluar pH and p*K*_*c*_ = −log_10_*k*_*c*_, we then have ${\text{pH}}_{0}=\text{p}K_{c}+{\text{log}}_{10}\left({\left[{\text{HCO}}_{3}^{-}\right]}_{\text{aq}}^{0}/{\left[{\text{CO}}_{2}\right]}_{\text{aq}}^{0}\right)$, where [CO_2_]_aq_ = [CO_2_${]}_{\text{aq}}^{0}$ since CO_2_ can move freely across the cell membrane (Vallés et al., 2015). For the intracellular domain, we have

(2) $$\begin{array}{l} \text{pH}-\text{p}K_{c}={\text{log}}_{10}\frac{{\left[{\text{HCO}}_{3}^{-}\right]}_{\text{aq}}}{{\left[{\text{CO}}_{2}\right]}_{\text{aq}}}, \end{array}$$

where $\text{pH}=-{\text{log}}_{10}{\left[{\text{H}}^{+}\right]}_{\text{aq}}$ is the intracelluar pH. The cell contains various buffer solutions. In general, the chemical reaction for the intracellular buffer solution can be written as ${\text{H}}_{\ell }\text{Buf}\left(\text{aq}\right)⇌\ell {\text{H}}^{+}\left(\text{aq}\right)+{\text{Buf}}^{\ell -}\left(\text{aq}\right)$, where ℓ = 1, 2, 3, … for different buffer species and *Z*_Buf_ = −ℓ is the valence of the unprotonated buffer. The reaction equilibrium constant is similarly $k_{B,\ell }={\left[{\text{Buf}}^{\ell -}\right]}_{\text{aq}}{\left[{\text{H}}^{+}\right]}_{\text{aq}}^{\ell }/{\left[{\text{H}}_{\ell }\text{Buf}\right]}_{\text{aq}}$. With p*K*_*B*,ℓ_ = −log_10_*k*_*B*,ℓ_, we have $\ell \text{pH}-\text{p}K_{B,\ell }={\text{log}}_{10}\left({\left[{\text{Buf}}^{\ell -}\right]}_{\text{aq}}/{\left[{\text{H}}_{\ell }\text{Buf}\right]}_{\text{aq}}\right)$. Without loss of generality, we lump all buffer solutions into one and use ℓ, or equivalently *Z*_Buf_, as a model parameter. By default, we let ℓ = 1 and simplify the notation of p*K*_*B*,ℓ_, Buf^ℓ−^, and H_ℓ_Buf as p*K*_*B*_, Buf^−^, and HBuf, respectively. We will discuss the impact of ℓ later.

The conservation equation for the non-reactive ion species is

(3) $$\begin{array}{l} \frac{d}{dt}\left(Vc_{n}\right)=4\pi r^{2}J_{n},\;n\in \left\{{\text{Na}}^{+},{\text{K}}^{+},{\text{Cl}}^{-}\right\}, \end{array}$$

where *J*_*n*_ is the total ion flux across the membrane for each species and is determined by the boundary conditions of ion fluxes through the membrane channels, transporters, and pumps; *V* = 4π*r*^3^/3 is the cell volume. The conservation equation for the intracellular reactive species is

(4) $$\begin{array}{l} \frac{d}{dt}\left(Vc_{\text{H}}\right)-4\pi r^{2}J_{\text{H}}=\frac{d}{dt}\left(Vc_{\text{HC}{\text{O}}_{3}}\right)-4\pi r^{2}J_{\text{HC}{\text{O}}_{3}}+\frac{d}{dt}\left(Vc_{\text{Buf}}\right). \end{array}$$

The total flux for each ionic species is the sum of the fluxes through the relevant channels, transports, and pumps:

(5) $$\begin{array}{l} J_{\text{Na}}=J_{\text{Na},p}+J_{\text{NKCC},\text{Na}}+J_{\text{NKE},\text{Na}}+J_{\text{NHE},\text{Na}}+J_{\text{NBC},\text{Na}}, \end{array}$$

(6) $$\begin{array}{l} J_{\text{K}}=J_{\text{K},p}+J_{\text{NKCC},\text{K}}+J_{\text{NKE},\text{K}}, \end{array}$$

(7) $$\begin{array}{l} J_{\text{Cl}}=J_{\text{Cl},p}+J_{\text{NKCC},\text{Cl}}+J_{\text{AE}2,\text{Cl}}, \end{array}$$

(8) $$\begin{array}{l} J_{\text{H}}=J_{\text{NHE},\text{H}}, \end{array}$$

(9) $$\begin{array}{l} J_{{\text{HCO}}_{3}}=J_{{\text{AE}2,\text{HCO}}_{3}}+J_{{\text{NBC},\text{HCO}}_{3}}. \end{array}$$

The passive ion fluxes, *J*_*n,p*_, are proportional to the electrochemical potential difference of ions across the membrane (Li et al., 2015) and are typically mechanosensitive (Martinac, 2004),

(10) $$\begin{array}{l} J_{n,p}=\alpha_{n,p}G_{m}\left(RT\ln\;\Gamma_{n}-z_{n}FV_{m}\right),\;n\in \left\{{\text{Na}}^{+},{\text{K}}^{+},{\text{Cl}}^{-}\right\} \end{array}$$

where *V*_*m*_ is the membrane potential; *z*_*n*_ is the valence of each ionic species; $\Gamma_{n}=c_{n}^{0}/c_{n}$ is the ratio of extra- to intra-cellular ion concentrations; α_*n,p*_ is the permeability coefficient of each species, which depends on the channel property and the density of the channels in the membrane; *G*_*m*_ ∈ (0, 1) is a mechanosensitive gating function that generally follows a Boltzmann distribution, i.e., $G_{m}={\left[1+e^{-\beta_{1}\left(\tau_{m}-\beta_{2}\right)}\right]}^{-1}$, where β_1_ and β_2_ are two constants and τ_*m*_ is the cortical tension given by τ_*m*_ = σ*h*.

Since NKCC is mainly a passive transport, the flux through it can be expressed as (Bennett et al., 2008)

(11) $$\begin{array}{l} J_{\text{NKCC}}=J_{\text{NKCC},\text{Na}}=J_{\text{NKCC},\text{K}}=\frac{1}{2}J_{\text{NKCC},\text{Cl}}\\ \;=\alpha_{\text{NKCC}}RT\left(\ln\Gamma_{\text{Na}}+\ln\Gamma_{\text{K}}+2\ln\Gamma_{\text{Cl}}\right), \end{array}$$

where α_NKCC_ is the permeability coefficient independent of the cortical tension. Given the dependence of NHE on pH, the flux of NHE is expressed as

(12) $$\begin{array}{l} J_{\text{NHE}}=J_{\text{NHE},\text{Na}}=-J_{\text{NHE},\text{H}}=\alpha_{\text{NHE}}G_{\text{NHE}}RT\left(\ln\Gamma_{\text{Na}}-\ln\Gamma_{\text{H}}\right), \end{array}$$

where α_NHE_ is the permeability coefficient which does not significantly depend on cortical tension (Pang et al., 2012) and we assume it as constant. $G_{\text{NHE}}={\left[1+e^{\beta_{5}\left(\text{pH}-\beta_{6}\right)}\right]}^{-1}$ is a pH-gated function indicating the dependence of the NHE activity on pH (Figure 1B). Similarly, the flux through AE2 takes the form

(13) $$\begin{array}{l} J_{\text{AE2}}=J_{\text{AE}2,\text{Cl}}=-J_{{\text{AE}2,\text{HCO}}_{3}}=\alpha_{\text{AE2}}G_{\text{AE}2}RT\left(\ln\Gamma_{\text{Cl}}-\ln\Gamma_{{\text{HCO}}_{3}}\right), \end{array}$$

where α_AE2_ is the permeability coefficient of AE2 and is assumed to be independent of the cortical tension. $G_{\text{AE}2}={\left[1+e^{-\beta_{7}\left(\text{pH}-\beta_{8}\right)}\right]}^{-1}$ is a pH-gated function indicating the dependence of the AE2 activity on pH (Figure 1B). The fluxes through NBC depends on the combined electrochemical potential of the two species, i.e.,

(14) $$\begin{array}{l} J_{\text{NBC}}=J_{\text{NBC},\text{Na}}=\frac{J_{{\text{NBC},\text{HCO}}_{3}}}{n_{\text{NBC}}}\\ \;=\alpha_{\text{NBC}}\left[RT\ln\;\left(\Gamma_{\text{Na}}\Gamma_{{\text{HCO}}_{3}}^{n_{\text{NBC}}}\right)-\left(z_{\text{Na}}+n_{\text{NBC}}z_{{\text{HCO}}_{3}}\right)FV_{m}\right], \end{array}$$

where α_NBC_ is the permeability coefficient of NBC and *n*_NBC_ = 2 or 3 indicates the stoichiometry of NBC. In the paper, we will take *n*_NBC_ = 2 in our simulation. Since the NKE is voltage-dependent and its flux saturates at high concentration limits, we model the flux of Na^+^ and K^+^ through the Na^+^/K^+^ pump as

(15) $$\begin{array}{l} J_{\text{NKE}}=J_{\text{NKE},\text{Na}}=-\frac{3}{2}J_{\text{NKE},\text{K}}\\ \;=-\alpha_{\text{NKE}}G_{\text{V},\text{NKE}}{\left(1+\beta_{\text{NKE},\text{Na}}\Gamma_{\text{Na}}\right)}^{-3}{\left(1+\beta_{\text{NKE},\text{K}}/\Gamma_{\text{K}}\right)}^{-2}, \end{array}$$

where α_NKE_ is the permeability coefficient of the pump depending on the density of the pump as well as the concentration of ATP. β_NKE,Na_ and β_NKE,K_ are constants that scale Γ_Na_ and Γ_K_, respectively. The exponents 3 and 2 are the Hill's coefficients of Na^+^ and K^+^, respectively. Equation 15 ensures that the flux is zero when either 1/Γ_Na_ or Γ_K_ approaches zero; the flux saturates if 1/Γ_Na_ and Γ_K_ approaches infinity. *G*_V,NKE_ captures the voltage-dependence of the pump activity (Gadsby et al., 1985), $G_{\text{V},\text{NKE}}=2{\left[1+e^{-\beta_{3}\left(V_{m}-\beta_{4}\right)}\right]}^{-1}-1$, where β_3_ and β_4_ are constants (Figure 1C).

The electro-neutral condition should be satisfied for both the intracellular and the extracellular spaces. The condition for the intracellular space is maintained by enforcing ∑*z*_*n*_*c*_*n*_ = 0. The full set of equations for the system is as follows,

(16) $$\begin{array}{l} \frac{dr}{dt}=J_{\text{water}}=-\alpha_{\text{w}}\left[\frac{2h\sigma }{r}-RT\left(\sum c_{n}-\sum c_{n}^{0}\right)\right], \end{array}$$

(17) $$\begin{array}{l} \sum z_{n}c_{n}=0, \end{array}$$

(18) $$\begin{array}{l} \frac{d}{dt}\left(Vc_{\text{Na}}\right)=4\pi r^{2}\left(J_{\text{Na},p}+J_{\text{NKCC}}+J_{\text{NKE}}+J_{\text{NHE}}+J_{\text{NBC}}\right), \end{array}$$

(19) $$\begin{array}{l} \frac{d}{dt}\left(Vc_{\text{K}}\right)=4\pi r^{2}\left(J_{\text{K},p}+J_{\text{NKCC}}-\frac{2}{3}J_{\text{NKE}}\right), \end{array}$$

(20) $$\begin{array}{l} \frac{d}{dt}\left(Vc_{\text{Cl}}\right)=4\pi r^{2}\left(J_{\text{Cl},p}+2J_{\text{NKCC}}+J_{\text{AE2}}\right), \end{array}$$

(21) $$\begin{array}{l} \frac{d}{dt}\left(Vc_{\text{H}}\right)+4\pi r^{2}J_{\text{NHE}}=\frac{d}{dt}\left(Vc_{\text{HC}{\text{O}}_{3}}\right)\\ +4\pi r^{2}\left(J_{\text{AE2}}-n_{\text{NBC}}J_{\text{NBC}}\right)+\frac{d}{dt}\left(Vc_{\text{Buf}}\right). \end{array}$$

In this work, the given quantities (known model inputs) are: $c_{\text{Na}}^{0}$, $c_{\text{K}}^{0}$, $c_{\text{Cl}}^{0}$, $c_{\text{HC}{\text{O}}_{3}}^{0}$, *N*_*A*_, *P*_CO2_, and *N*_HBuf_ + *N*_Buf_. The six equations (Equations 16–21) are used to solve for the six unknowns: *r*, *V*_*m*_, *c*_Na_, *c*_K_, *c*_Cl_, and pH. The rest of the quantities are derived either from the given quantities or from the unknowns.

For steady-state response, Equations (16–21) can be directly implemented to solve for the six unknowns $\text{X}={\left(r,V_{m},c_{\text{Na}},c_{\text{K}},c_{\text{Cl}},\text{pH}\right)}^{T}$ by letting *d*/*dt* = 0. The membrane potential, *V*_*m*_, is solved by Equation (17). To solve for the cell transient response to sudden changes, since Equation (17) does not explicitly contain *V*_*m*_, it does not give the time-dependent *V*_*m*_ in its original form. We will thus rewrite Equation (17) as follows. At each time step, we have $\sum z_{n}c_{n}=0\Rightarrow \sum z_{n}Vc_{n}=0\Rightarrow \sum z_{n}d\left(Vc_{n}\right)/dt=0\Rightarrow \sum z_{n}4\pi r^{2}J_{n}=0$, which explicitly contains *V*_*m*_ and will replace Equation (17) in solving for cell transient responses. In this case, the initial condition for the cell volume should give rise to the concentrations of *c*_*A*_ and *c*_Buf_ such that ∑*z*_*n*_*c*_*n*_ = 0 is satisfied.

### 2.2. Brief Model Analysis

Here we will provide some brief analysis that provide useful insights on the responses of the cell. At steady state, all the time-depend terms in Equations (16–21) vanish and the system becomes

(22) $$\begin{array}{l} \frac{2h\sigma }{r}-RT\left(\sum c_{n}-\sum c_{n}^{0}\right)=0, \end{array}$$

(23) $$\begin{array}{l} \sum z_{n}c_{n}=0, \end{array}$$

(24) $$\begin{array}{l} 0=J_{\text{Na},p}+J_{\text{NKCC}}+J_{\text{NKE}}+J_{\text{NHE}}+J_{\text{NBC}}, \end{array}$$

(25) $$\begin{array}{l} 0=J_{\text{K},p}+J_{\text{NKCC}}-\frac{2}{3}J_{\text{NKE}}, \end{array}$$

(26) $$\begin{array}{l} 0=J_{\text{Cl},p}+2J_{\text{NKCC}}+J_{\text{AE2}}, \end{array}$$

(27) $$\begin{array}{l} 0=J_{\text{NHE}}-J_{\text{AE2}}+n_{\text{NBC}}J_{\text{NBC}}. \end{array}$$

We first observe that the permeability coefficient of water, α_w_, is not present in the steady-state solution, suggesting that the steady-state cell volume variation is independent of the expression level of AQPs, although AQPs are important for water flux. We will discuss the role of AQP in the result sections.

Among the six equations, only Equations (22, 23) explicitly contain *r* (e.g., *c*_*A*_ = *N*_*A*_/*V*), and Equations (24–27) are ion fluxes as functions of ion concentration, membrane potential, and pH. We can therefore find an equation that solves for the cell volume. Multiplying Equation (23) with *RT* and adding it to Equation (22) provides a cubic equation in *r*. To perform a scaling analysis, we let *R*_0_ be a generic scale of cell radius. The cell radius can thus be written in a non-dimensional form on the order of one: $\bar{r}=r/R_{0}$. The cubic equation becomes

(28) $$\begin{array}{l} p_{3}{\bar{r}}^{3}+p_{2}{\bar{r}}^{2}-p_{0}=0,\\ p_{3}=\left(\sum c_{n}^{0}-2c_{\text{Cl}}-2c_{{\text{HCO}}_{3}}\right)R_{0}^{3},\;p_{2}=\frac{2h\sigma }{RT}R_{0}^{2},\\ p_{0}=\frac{3}{4\pi }\left[\frac{2+10^{\left({\text{pK}}_{\text{B}}-\text{pH}\right)}}{1+10^{\left({\text{pK}}_{\text{B}}-\text{pH}\right)}}\left(N_{\text{HBuf}}+N_{\text{Buf}}\right)+2N_{A}\right], \end{array}$$

where *p*_3_, *p*_2_, *p*_0_ > 0 and are all in dimension of moles. Denote $\Delta c=\sum c_{n}-\sum c_{n}^{0}$ as the osmolarity difference across the cell membrane. We thus have $\left(\sum c_{n}^{0}-2c_{\text{Cl}}-2c_{{\text{HCO}}_{3}}\right)=2c_{\text{Buf}}+c_{\text{HBuf}}+2c_{\text{A}}-\Delta c$. Under physiological-relevant conditions, (2*c*_Buf_ + *c*_HBuf_ + 2*c*_A_ − Δ*c*) is on the order of 100 mM, *R*_0_ is on the order of 10 μm, *h* is on the order of 10^−7^ m, σ is on the order of 1 kPa, *N*_HBuf_ + *N*_Buf_ and *N*_*A*_ are on the order of 0.1 pmol, and $10^{\left(\text{pH}-{\text{pK}}_{\text{B}}\right)}$ is on the order of 1. Together, $p_{3}\sim 10^{-1}\;\text{pmol}$, $p_{2}\sim 10^{-5}\;\text{pmol}$, $p_{0}\sim 10^{-1}\;\text{pmol}$. If the product of *hσ* increases by one order of magnitude, *p*_2_ will also increase by one order of magnitude. Nevertheless, we have $4p_{2}^{3}/27p_{3}^{2}<p_{0}$, which indicates that Equation (28) has one unique real solution for $\bar{r}$. Although the system described by Equations (22–27) is highly nonlinear, when the cell reaches steady state, the cell volume is uniquely determined. We can further find an approximate analytical solution for $\bar{r}$ using the perturbation method. Since *p*_2_ ≪ *p*_3_ and *p*_2_ ≪ *p*_0_, let ϵ = *p*_2_ be a small parameter for the equation $p_{3}{\bar{r}}^{3}+\epsilon {\bar{r}}^{2}-p_{0}=0$. The solution for $\bar{r}$ is expanded as $\bar{r}={\bar{r}}_{0}+\epsilon \bar{r}+\epsilon^{2}{\bar{r}}^{2}+\cdots \;$. The ϵ^0^-order of equation solves for ${\bar{r}}_{0}$ as ${\bar{r}}_{0}={\left(p_{0}/p_{1}\right)}^{1/3}$ and the ϵ^1^-order of equation solves for ${\bar{r}}_{1}$ as ${\bar{r}}_{1}=-1/\left(3p_{3}\right)$. The solution for $\bar{r}$ is thus approximately

(29) $$\begin{array}{l} \bar{r}≃\underset{\epsilon^{0}-\text{order}}{\underset{︸}{{\left(\frac{p_{0}}{p_{3}}\right)}^{1/3}}}\underset{\epsilon^{1}-\text{order}}{\underset{︸}{-\frac{p_{2}}{3p_{3}}}}. \end{array}$$

From the order of magnitude analysis, we can see that *c*_*n*_ and pH are the primary variables determining the cell volume. σ has an important but secondary effect. α_w_ does not influence the cell volume at a steady state. The factor 2*hσ*/*R*_0_ in *p*_2_ can be rewritten as $2h\sigma /R_{0}=\left(2h\sigma /r\right)\left(r/R_{0}\right)=\bar{r}\Delta p=\bar{r}RT\Delta c$. We then have $p_{2}/p_{3}=\bar{r}\Delta c/\left(2c_{\text{Buf}}+c_{\text{HBuf}}+2c_{\text{A}}-\Delta c\right)\sim 10^{-4}$ based on the orders of *p*_2_ and *p*_3_. Since $\bar{r}$ is on the order of 1, then $\Delta c/\left(2c_{\text{Buf}}+c_{\text{HBuf}}+2c_{\text{A}}-\Delta c\right)\sim 10^{-4}$. Therefore, $\left(2c_{\text{Buf}}+c_{\text{HBuf}}+2c_{\text{A}}-\Delta c\right)\sim 10^{4}\Delta c$ and 2*c*_Buf_ + *c*_HBuf_ + 2*c*_A_ − Δ*c* ≃ 2*c*_Buf_ + *c*_HBuf_ + 2*c*_A_. The sum (2*c*_Buf_ + *c*_HBuf_ + 2*c*_A_) is typically on the order of 100 mM, suggesting that Δ*c* is on the order of 0.01 mM. Given the large overall osmolarity of the intracellular and extracellular environments, which is on the order of 100 mM, the Δ*c* is negligible and is often approximated as zero (Mori, 2012). Equation 29 can further be simplified as

(30) $$\begin{array}{l} \bar{r}≃(\frac{3}{4\pi R_{0}^{3}\left(2c_{\text{Buf}}+c_{\text{HBuf}}+2c_{\text{A}}\right)}\times \\ {\left[\frac{2+10^{\left({\text{pK}}_{\text{B}}-\text{pH}\right)}}{1+10^{\left({\text{pK}}_{\text{B}}-\text{pH}\right)}}\left(N_{\text{HBuf}}+N_{\text{Buf}}\right)+2N_{A}\right])}^{1/3}\\ \;-\frac{2h\sigma }{3RTR_{0}\left(2c_{\text{Buf}}+c_{\text{HBuf}}+2c_{\text{A}}\right)}. \end{array}$$

The leading order solution is

(31) $$\begin{array}{l} {\bar{r}}_{0}≃(\frac{3}{4\pi R_{0}^{3}\left(2c_{\text{Buf}}+c_{\text{HBuf}}+2c_{\text{A}}\right)}\times \\ \;\left[\frac{2+10^{\left({\text{pK}}_{\text{B}}-\text{pH}\right)}}{1+10^{\left({\text{pK}}_{\text{B}}-\text{pH}\right)}}\left(N_{\text{HBuf}}+N_{\text{Buf}}\right)+2N_{A}\right]{)}^{1/3}. \end{array}$$

On the leading order, the cell volume is

(32) $$\begin{array}{l} V=\frac{4}{3}\pi r^{3}=\frac{4}{3}\pi {\bar{r}}^{3}R_{0}^{3}≃\frac{2+10^{\left({\text{pK}}_{\text{B}}-\text{pH}\right)}}{1+10^{\left({\text{pK}}_{\text{B}}-\text{pH}\right)}}\frac{\left(N_{\text{HBuf}}+N_{\text{Buf}}\right)}{\left(2c_{\text{Buf}}+c_{\text{HBuf}}+2c_{\text{A}}\right)}\\ \;+2\frac{N_{A}}{\left(2c_{\text{Buf}}+c_{\text{HBuf}}+2c_{\text{A}}\right)}. \end{array}$$

Equation 32 shows that the cell volume is linear in *N*_HBuf_ + *N*_Buf_ and *N*_*A*_ due to the assumed impermeability of the buffer solution and proteins in the model (Kay, 2017; Yellin et al., 2018). Different cells may have different amounts and types of buffer solutions and proteins. pH plays an important role in cell volume regulation; cells' ability to control pH also affects cells' ability to control volume. The concentration of permeable ion species can be expressed in terms of impermeable species, *c*_Buf_, *c*_HBuf_, and *c*_A_. Of note, Equation (32) is not a result of Equations (24–27) and is thus independent of the choice of ion channels, transporters, and pumps. Equations 32 does depend on the spherical assumption we have made on the cell. In this work, we will limit our study on the volume response of spherical cells under perturbations.

Equations 24 to 27 can be scaled by an arbitrary constant. Without loss of generality, we can choose the passive K^+^ channel permeability coefficient, α_K,*p*_, as the overall permeability scale, α_0_, of ion dynamics, i.e., α_0_ = α_K,*p*_. We will then rescale all the other permeability coefficients by α_0_, which is equivalent to rescaling each flux, *j*_*n,p*_ = *J*_*n,p*_/α_0_, *j*_NKE_ = *J*_NKE_/α_0_, *j*_NKCC_ = *J*_NKCC_/α_0_, *j*_NHE_ = *J*_NHE_/α_0_, *j*_AE2_ = *J*_AE2_/α_0_, *j*_NBC_ = *J*_NBC_/α_0_. Equations 24 to 27 becomes

(33) $$\begin{array}{l} 0=j_{\text{Na},p}+j_{\text{NKCC}}+j_{\text{NKE}}+j_{\text{NHE}}+j_{\text{NBC}}, \end{array}$$

(34) $$\begin{array}{l} 0=j_{\text{K},p}+j_{\text{NKCC}}-\frac{2}{3}j_{\text{NKE}}, \end{array}$$

(35) $$\begin{array}{l} 0=j_{\text{Cl},p}+2j_{\text{NKCC}}+j_{\text{AE2}}, \end{array}$$

(36) $$\begin{array}{l} 0=j_{\text{NHE}}-J_{\text{AE2}}+n_{\text{NBC}}j_{\text{NBC}}, \end{array}$$

which provide the same solution as Equations (24–27). Therefore, the overall permeability scale, α_0_, is a free parameter for the steady-state problem and only affects the time scale of the transient response before the system reaches steady-state. For steady-state solution, the relative values of the permeability coefficients of each species will play the major role in determining the cell response, including the cell volume as seen in Equation (29).

### 2.3. Parameters

Most of the model parameters can be estimated or obtained from the literature. The overall permeability scale, α_0_, is chosen such that the predicted transient response upon perturbation lasts about 10 min before the system reaches a steady state. This time scale comes from both our experimental observation when cells typically research steady-state after perturbation (Yellin et al., 2018) and the possible magnitude of fluxes from the average number of ion channels, transporters, or pumps per unit cell surface area (Gadsby, 2009). The relative permeability coefficients of various ion channels, transporters, and pumps, such as α_NKE_/α_0_, α_NKCC_/α_0_, α_NHE_/α_0_, and α_AE2_/α_0_, are fitted such that the model output vector, $\text{X}={\left(r,V_{m},c_{\text{Na}},c_{\text{K}},c_{\text{Cl}},\text{pH}\right)}^{T}$, is consistent with physiological-relevant numbers reported in the literature. For example, the intracellular concentrations of sodium, potassium, and chloride are on the order of 5 mM, 150 mM, and 10–50 mM, respectively. The typical membrane potential is about −65 mV and the intracellular pH is about 7.4. We then solve an inverse problem in which **X** is given and the relative permeability coefficients of various ion channels, transporters, and pumps are unknowns. The solution for the unknowns determines the order of magnitude for α_NKE_, α_NKCC_, α_NHE_, and α_AE2_. The value for α_NBC_ is estimated and in this model we allow it to vary across several orders of magnitudes. This variation can come from multiple mechanisms, including regulation via calcium (Yamada et al., 1996; Ruiz et al., 1999; Bachmann et al., 2006) and biochemical signals (Ruiz et al., 1996, 1998).

In the model, we consider two chemical reactions: the bicarbonate-carbonic acid pair and the buffer solution. The buffer solution is generic and contains all possible buffer mechanisms, including the phosphate buffer (Thomas and O'Shea, 2005). The p*K*_*B*_ for the intracellular buffer is based on values in literature (Weinstein, 1986). Since different cell types have a different amount of buffer solution, the value of p*K*_*B*_ is likely to be cell-type dependent. Since cells can modulate their intracellular biochemical environment such that NHE inhibition does not change the membrane potential (Mahnensmith and Aronson, 1985), we therefore adjust p*K*_*B*_ in a way that its value ensures that NHE has a negligible effect on the membrane potential.

The full parameters used in the model are provided in Table 1. These parameters will not change throughout the simulation unless otherwise specified or varied. Most of the parameter choice is generic and does not qualitatively affect the results and conclusions. We instead focus more on the scaling laws of the system and draw a general conclusion on cell volume regulation. Matlab^Ⓡ^ was used to program and solve the equations. The roots of the non-linear equations at each time step were found by the Newton-Raphson Iteration method (Ben-Israel, 1966).

**Table 1:** Model parameters.

**Parameter**	**Description**	**Value**	**Source**
*h* (nm) ^*^	Cortical thickness	500	Yellin et al., 2018
*R* (J/mol/K)	Ideal gas constant	8.31	Physical constant
*T* (K)	Absolute temperature	310	Physiological condition
σ_*a*_ (Pa) ^*^	Active contraction	10^3^	Yellin et al., 2018
*N*_*A*_ (mol) ^*^	Total intracellular A^−^	0.142	Estimated
*N*_Bul_+*N*_HBul_ (pmol) ^*^	Total intracellular Buffer Solution	0.16	Estimated
α_w_ (m/Pa/s) ^*^	Permeability coefficient of water (Equation 1)	10^−10^	Jiang and Sun, 2013
α_0_ (mol^2^/J/m^2^/s) ^*^	Overall ion flux rate scale	5 ×10^−9^	See text
α_Na, *p*_ (mol^2^/J/m^2^/s) ^*^	Permeability coefficient of Na channel (Equation 10)	0.05α_0_	Fitted (see text)
α_K,*p*_ (mol^2^/J/m^2^/s) ^*^	Permeability coefficient of K channel (Equation 10)	α_0_	See text
α_Cl, *p*_ (mol^2^/J/m^2^/s) ^*^	Permeability coefficient of Cl channel (Equation 10)	0.1α_0_	Fitted (see text)
α_NKE_ (mol/m^2^/s) ^*^	Permeability coefficient of NKE (Equation 15)	$10^{5}\alpha_{0}/RT$	Fitted (see text)
α_NKCC_ (mol^2^/J/m^2^/s) ^*^	Permeability coefficient of NKCC (Equation 11)	$10^{-5}\alpha_{0}$	Fitted (see text)
α_NHE_ (mol^2^/J/m^2^/s) ^*^	Permeability coefficient of NHE (Equation 12)	$10^{-1}\alpha_{0}$	Fitted (see text)
α_AE2_ (mol^2^/J/m^2^/s) ^*^	Permeability coefficient of AE2 (Equation 13)	0.06α_0_	Fitted (see text)
α_NBC_ (mol^2^/J/m^2^/s) ^*^	Permeability coefficient of NBC (Equation 14)	$10^{-2}\alpha_{0}$	Fitted (see text)
β_NKE, Na_ ^†^	Constant in *J*_NKE_ (Equation 15)	0.1	Estimated
β_NKE, K_ ^†^	Constant in *J*_NKE_ (Equation 15)	10	Estimated
β_1_ (m/N) ^†^	In $G_{m}={\left[1+e^{-\beta_{1}\left(\tau_{m}-\beta_{2}\right)}\right]}^{-1}$ (Equation 10)	2 ×10^3^	Estimated
β_2_ (N/m) ^†^	In $G_{m}={\left[1+e^{-\beta_{1}\left(\tau_{m}-\beta_{2}\right)}\right]}^{-1}$ (Equation 10)	5 ×10^−4^	Estimated
β_3_ (1/mV) ^†^	In $G_{\text{V},\text{NKE}}=2/\left[1+e^{-\beta_{3}\left(V_{m}-\beta_{4}\right)}\right]-1$ (Equation 15)	0.03	Gadsby et al., 1985
β_4_ (mV) ^†^	In $G_{\text{V},\text{NKE}}=2/\left[1+e^{-\beta_{3}\left(V_{m}-\beta_{4}\right)}\right]-1$ (Equation 15)	−150	Gadsby et al., 1985
β_5_ ^†^	In $G_{\text{NHE}}={\left[1+e^{\beta_{5}\left(\text{pH}-\beta_{6}\right)}\right]}^{-1}$ (Equation 12)	15	Casey et al., 2010
β_6_ ^†^	In $G_{\text{NHE}}={\left[1+e^{\beta_{5}\left(\text{pH}-\beta_{6}\right)}\right]}^{-1}$ (Equation 12)	7.2	Casey et al., 2010
β_7_ ^†^	In $G_{\text{AE}2}={\left[1+e^{-\beta_{7}\left(\text{pH}-\beta_{8}\right)}\right]}^{-1}$ (Equation 13)	10	Casey et al., 2010
β_8_ ^†^	In $G_{\text{AE}2}={\left[1+e^{-\beta_{7}\left(\text{pH}-\beta_{8}\right)}\right]}^{-1}$ (Equation 13)	7.1	Casey et al., 2010
*k*_*H*_ (atm/M)	Henry's constant	29	Weinstein, 1986
*P*_C_O__2__ (atm)	Partial pressure of CO_2_	5%	Physiological condition
p*K*_*c*_	p*K* for bicarbonate-carbonic acid pair (Equation 2)	6.1	Weinstein, 1986
p*K*_*B*_ ^*^	p*K* for intracellular buffer	6.7	See text
$c_{\text{Na}}^{0}$ (mM)	Na^+^ concentration in the medium	145	Physiological condition
$c_{\text{K}}^{0}$ (mM)	K^+^ concentration in the medium	9	Physiological condition
$c_{\text{Cl}}^{0}$ (mM)	Cl^−^ concentration in the medium	105	Physiological condition
$c_{\text{HC}{\text{O}}_{3}}^{0}$ (mM)	${\text{HCO}}_{3}^{-}$ concentration in the medium	35	Physiological condition
$c_{\text{G}}^{0}$ (mM)	Large molecular concentration in the medium	25	Physiological condition

* *Cell-type dependent*.

† *Intrinsic properties of channels, transporters, and pumps (cell-type independent)*.

## 3. Results and Discussion

### 3.1. Transient Water Flux and Intracellular Osmolarity

Given the importance of NHE in cellular function, volume regulation, and its coupling to AE2 through the bicarbonate-carbonic acid pair, we will start with NHE as an example to illustrate the transient response of the cell upon channel inhibition. In a theoretical experiment, the inhibition of NHE is achieved by reducing the permeability coefficient of NHE, i.e., α_NHE_. In a laboratory experiment, NHE inhibition can be achieved by adding inhibitors such as EIPA or by interfering with the expression of NHE using techniques such as siRNA, shRNA, or CRISPR/cas9. Here we use a 10^3^-fold decrease in α_NHE_ to represent a generic inhibition of the transporter; in later sections, we will discuss the effect of different levels of inhibition on the cell response.

**Figure 2:**
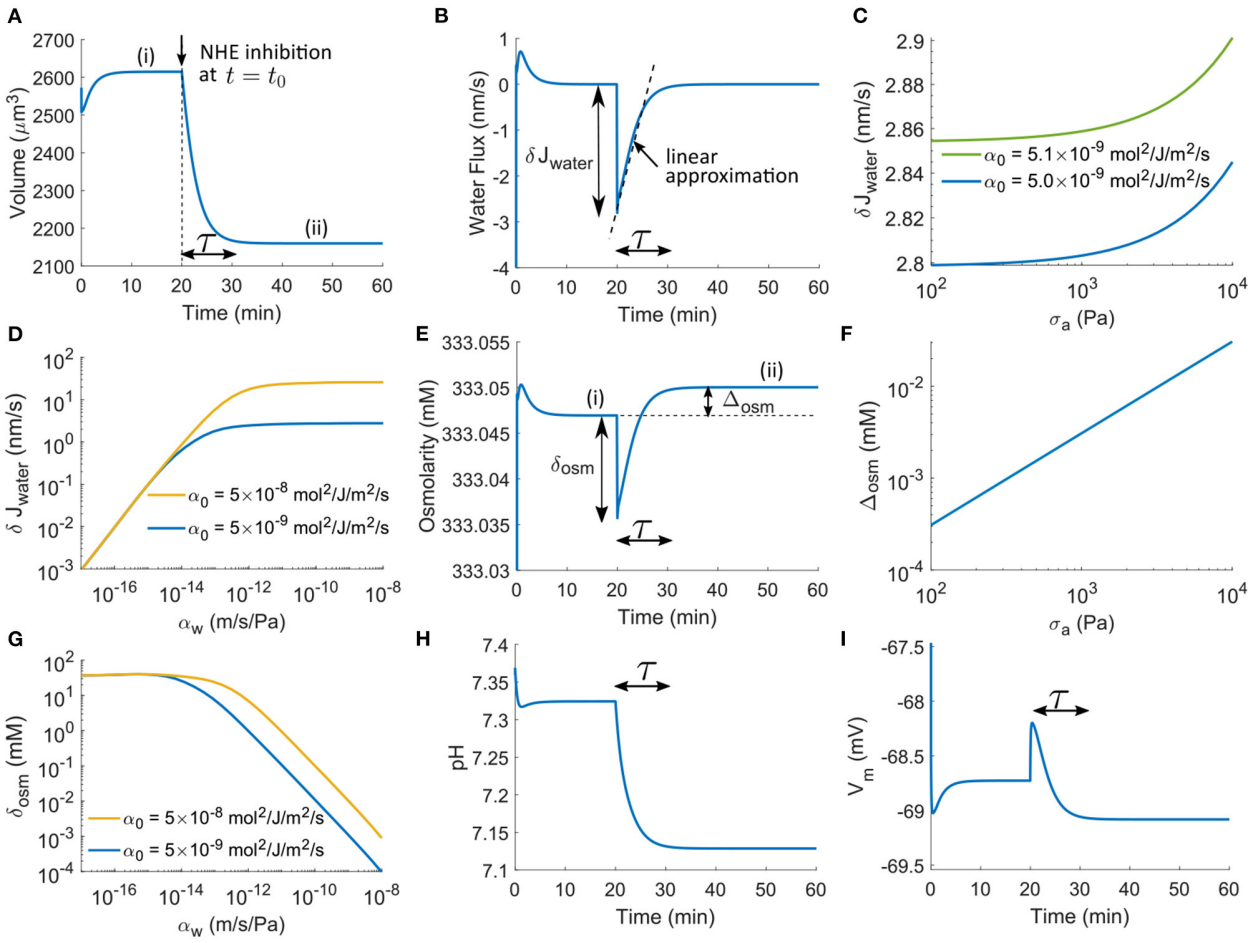
Model prediction on the cell transient response upon NHE inhibition applied at *t*_0_ = 20 min by reducing α_NHE_ by three orders of magnitude. **(A)** Transient response of cell volume as a function of time. τ is the time scale of the transient regime. **(B)** Transient response of cell water flux. **(C)** The maximum magnitude of the transient water flux upon NHE inhibition as a function of cortical stress, σ, for different α_0_. **(D)** The maximum magnitude of the transient water flux upon NHE inhibition as a function of the water permeability coefficient, α_w_, for different α_0_. **(E)** Transient response of intracellular osmolarity. The extracellular osmolarity is 333 mM. **(F)** The magnitude of the steady-state osmolarity difference before and after NHE inhibition as a function of cortical stress σ_*a*_. **(G)** The magnitude of the osmolarity difference before and after NHE inhibition as a function of α_w_ for different α_0_. **(H)** Transient response of the intracellular pH. **(I)** Transient response of the cell membrane potential.

We apply NHE inhibition at *t*_0_ = 20 min where the cell has reached a steady state (regime i) from an initial condition (Figure 2A). The cell volume is predicted to decrease upon inhibition (Figure 2A); this prediction will be different if the permeability coefficient of NBC increases, which will be discussed later. The time scale of the transient response, τ (Figure 2A), is mainly affected by the overall permeability scale, α_0_: τ decreases as α_0_ increases. After the transient regime, the cell reaches a steady state again (regime ii).

The cell volume decrease results from the water efflux during the transient regime (Figure 2B). Upon inhibition, an initial efflux with a maximum magnitude of δ*J*_water_ is seen, followed by a gradually decreasing efflux. The cell volume reduction corresponds to a change in cell radius, Δ*r*. Equation 1 suggests that $\Delta r=\int_{t_{0}}^{t_{0}+\tau }J_{\text{water}}dt$. If we approximate the water flux as a linear function in time during the time period τ (Figure 2B), we thus have Δ*r* ≈ (δ*J*_water_)τ/2. The maximum magnitude can then be expressed in terms of Δ*r* as δ*J*_water_ = 2Δ*r*/τ. Since Δ*r* is slightly affected by σ (see section Brief Model Analysis), so is δ*J*_water_ (Figure 2C). The model predicts that the increase of δ*J*_water_ is only < 2% even if σ has increased two orders of magnitude. Compared to σ, the overall permeability scale, α_0_, has a more significant effect: 2% increase of α_0_ leads to about 2% change of δ*J*_water_ (Figure 2C).

When α_w_ is in the physiological regime ($\alpha_{\text{w}}\sim 10^{-10}$ m/Pa/s), one order of magnitude increase of α_0_ leads to approximately one order of magnitude increase of δ*J*_water_ (Figure 2D, see the two lines). Although Δ*r* is independent of α_w_, δ*J*_water_ decreases with decreasing α_w_ when α_w_ is several orders of magnitude lower than the physiological-relevant regime, where δ*J*_water_ becomes independent of α_0_. However, within the physiological-relevant regime of α_w_, δ*J*_water_ remains as a constant (Figure 2D). The lipid membrane of mammalian cells is permeable to water with a permeability around $\alpha_{\text{w}}=1\times 10^{-12}$ m/s/Pa in the absence of the AQPs (Li et al., 2020). Thus, even if AQP is inhibited, this value of α_w_ will still lead to a permeability-independent δ*J*_water_ unless the overall permeability scale, α_0_, is two orders of magnitude higher than expected (Figure 2D). This result indicates that the limiting factor for the maximum water flux δ*J*_water_ depends on the relative coefficients of water and ion fluxes. Under normal conditions, water permeability is not the limiting factor. Together, the maximum magnitude of water flux across a cell membrane, in addition to the changes in solution concentration, is mainly affected by the overall permeability scale of ion channels, transporters, and pumps.

Water flux results from a hydrostatic pressure and osmotic pressure imbalance. The initial instantaneous water flux δ*J*_water_ comes from the initial osmolarity change δ_osm_ (Figure 2E). At steady states (i) and (ii) the two pressures are in balance. Define $\Delta_{\text{osm}}=\sum c_{n}^{\left(\text{ii}\right)}-\sum c_{n}^{\left(\text{i}\right)}$ as the difference between the osmolarity at the two steady states after and before NHE inhibition (Figure 2E). By Equation (22), we have

(37) $$\begin{array}{l} \Delta_{\text{osm}}=\frac{2h\sigma }{RT}\left(\frac{1}{r^{\left(\text{ii}\right)}}-\frac{1}{r^{\left(\text{i}\right)}}\right). \end{array}$$

Given that the original cell radius *r* is on the order of 8 μm and the change of radius is on the order of 0.5 μm, the term in the parenthesis is on the order of 10^−2^ μm^−1^. Besides,*h* is on the order of 0.5 μm, and σ is on the order of 10^3^ Pa. We thus find $\Delta_{\text{osm}}\sim 5\times 10^{-3}$ mM, which is <0.02‰ of the absolute value of the intracellular osmolarity, 333.05 mM (the extracellular constant osmolarity is 333 mM in this model). Therefore, the osmolarity difference across the cell membrane is tiny compared to the absolute osmolarity under consideration.

Although Δ_osm_ is proportional to σ by Equation (37), two orders of magnitude change of σ will still keep Δ_osm_ at a low level much <1 mM (Figure 2F). We thus conclude that before and after ion transporter perturbation, the intracellular osmolarity remains almost the same; the small change in osmolarity comes from the cortical tension. Of note, here we have assumed positive cortical tension; Δ_osm_ can be negative if the cortical tension becomes negative upon cell volume decrease. Since the effect from the cortical tension is much smaller than that from solute concentration, δ_osm_ can be approximated from Equation (16) as δ_osm_ ≈ δ*J*_water_/(α_w_*RT*) ≈ 2Δ*r*/(τα_w_*RT*), which indicates that the immediate change of osmolarity upon transporter perturbation is mainly determined by α_w_ and τ (i.e., α_0_). Interestingly, small water permeability or large overall permeability scale can increase δ_osm_ to the order of 1 mM (Figure 2G). Although α_w_ and α_0_ do not influence the cell when it reaches a steady state, it has a significant impact on the transient osmolarity. It has also been found that the water permeability can depend on membrane tension (Ozu et al., 2013; Bahari et al., 2020). With this consideration, the transient time scale will be different, i.e., the cell will reach a steady state either faster or slower depends on whether the cell expands or shrinks. This result also has implications on cell migration. When ion transporters are perturbed during cell migration, the expression level of AQPs and ion transporters will collectively determine the transient intracellular concentrations of ions, leading to a modulation of downstream signals that depend on the ionic content.

The pH and membrane potential changes (Figures 2H,I) show the same transient time scale before a steady state is reached. Inhibition of NHE acidifies the cell (Figure 2H) but does not affect the membrane potential (Figure 2I), both being consistent with our understanding of NHE (Boron and Boulpaep, 1989). The acidification comes from the reduced H^+^ extrusion upon NHE inhibition. We will have further discussion in the following sections on how this acidification leads to cell volume reduction.

### 3.2. Mechanisms of NHE in Cell Volume Regulation

It is attempting to think that the reduced intracellular sodium concentration is responsible for cell volume reduction upon NHE inhibition. This speculation comes from the following reasoning. NHE intakes one Na^+^ and removes one H^+^ at one time. The removed H^+^ goes with ${\text{HCO}}_{3}^{-}$ via AE2, a chloride and bicarbonate exchanger (Figure 1A). Since CO_2_ is permeable to the cell membrane, the cytoplasm can continuously produce H^+^ and ${\text{HCO}}_{3}^{-}$, which will be transported out of the cells. With the NHE in place, the cell would have a net gain of two ions at one time, namely Na^+^ and Cl^−^ (Lang et al., 1998; Vallés et al., 2015). Inhibition of NHE would reduce this net gain and thus decrease cell volume. Below we will explain why the reduced Na^+^ influx itself upon NHE inhibition is unlikely to be responsible for cell volume reduction. We will also provide a possible mechanism of NHE-induced cell volume decrease.

**Figure 3:**
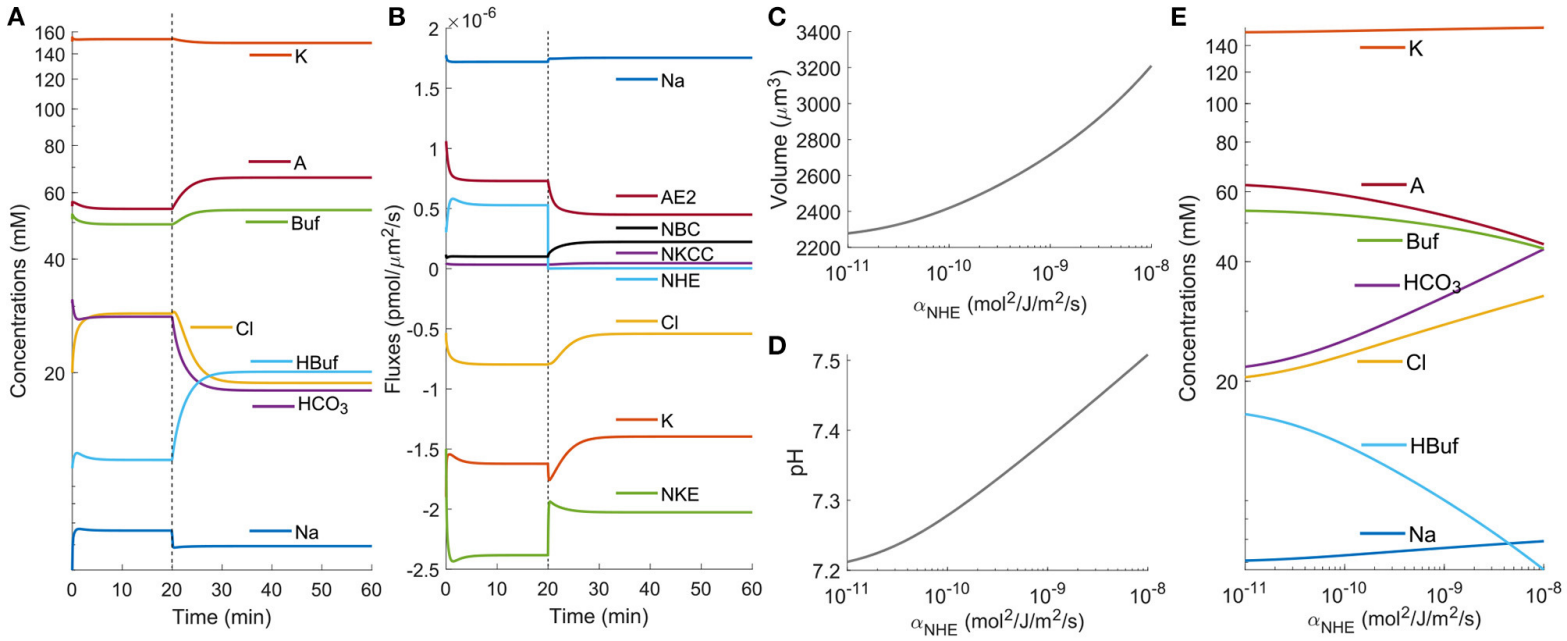
Model predictions of the cellular response upon NHE inhibition. **(A)** Intracellular ion concentrations upon NHE inhibition at *t*_0_ = 20 min. **(B)** Ion fluxes across each channel and co-transporter upon NHE inhibition at *t*_0_ = 20 min. **(C)** Model prediction of the cell volume when NHE is inhibited. **(D)** Model prediction of the intracellular pH when NHE is inhibited. **(E)** Model prediction of the intracellular ion concentrations when NHE is inhibited.

Figure 2B shows that the net cell volume change is a result of the integration of *J*_water_ over the time τ. A small instantaneous water flux δ*J*_water_ with negligible time span (*t* ≪ τ) is not able to generate significant volume change. We, therefore, infer that the instantaneous osmolarity fluctuation δ_osm_ does not lead to volume change, either. The transient osmolarity change over time τ (Figure 2E) is a combination of the concentration change of each ion species inside the cell (Figure 3A). The model predicts that among all the species under consideration, only Na^+^ experiences an instantaneous (*t* ≪ τ) concentration change while other species have a time scale of about τ (Figure 3A). This result suggests that the effect of NHE on Na^+^ is immediate and is thus not expected to contribute to the large volume change seen in Figure 2A. The model also predicts a significant concentration decrease for bicarbonate, ${\text{HCO}}_{3}^{-}$. This decrease is expected from the bicarbonate-carbonic acid pair reaction and is one of the main reasons behind the NHE inhibition-induced cell volume reduction.

Since NHE inhibition acidifies the cell, let's consider the following scaling analysis. If at the beginning the intracellular pH is 7.4, from Equation (2) we find the intracellular concentration of ${\text{HCO}}_{3}^{-}$ to be 33.5 mM. If after acidification the intracellular pH is reduced to 7.2, the ${\text{HCO}}_{3}^{-}$ concentration then becomes 21.1 mM. This ~10 mM change in concentration is significant enough to build up pressure difference to drive water flux, although we recognize that the final cell volume change is a combination of all the ion species, not ${\text{HCO}}_{3}^{-}$ alone. As a side note, 0.5 mM change in concentration corresponds to 1 kPa change in pressure (Li et al., 2020). A second significance with ${\text{HCO}}_{3}^{-}$ concentration reduction is that it reduces AE2 flux, bringing in fewer Cl^−^ as a result. The model also predicts a remarkable concentration decrease of Cl^−^ on the order of 10 mM (Figure 3A).

We also observed a concentration increase of A^−^, Buf^−^, and HBuf (Figure 3A). This trend is expected because these species are not permeable to the cell membrane, so when the cell volume decreases their concentrations increase. For permeable species, the concentration change is mainly a result of the flux change through the corresponding transporters (Figure 3B), except for the changes that come from the chemical reaction. The model predicts that for channels or transports that directly involve Na^+^, their transient flux changes are almost immediate (much less than the time scale τ). For example, we see a quick reduction in the magnitude of ion flux through NKE as a result of the reduced intracellular Na^+^ concentration. This change also leads to a slight reduction for the intracellular K^+^ (Figure 3A). To summarize, the total intracellular osmolarity change over time τ is a combined effect of the transient responses of ion fluxes and concentration modulation, especially the NHE and AE2 pair, not a direct result of Na^+^ decrease as has been postulated for a long time.

After the transient response, the cells reach a steady state. Figures 3C–E shows the steady-state cell volume, intracellular pH, and intracellular ion concentration of each species as a function of the permeability coefficient of NHE, α_NHE_. The results can be interpreted as different levels of NHE inhibition. For example, as NHE is progressively inhibited, the cell volume decreases continuously, corresponds to increased net water efflux. This result is consistent with NHE inhibition during cell migration that the cell speed decreases progressively as NHE is inhibited with increasing concentration of EIPA (Stroka et al., 2014) or HOE642 (Stock et al., 2005).

### 3.3. Implication of NBC on Cell Volume Regulation Upon NHE Inhibition

**Figure 4:**
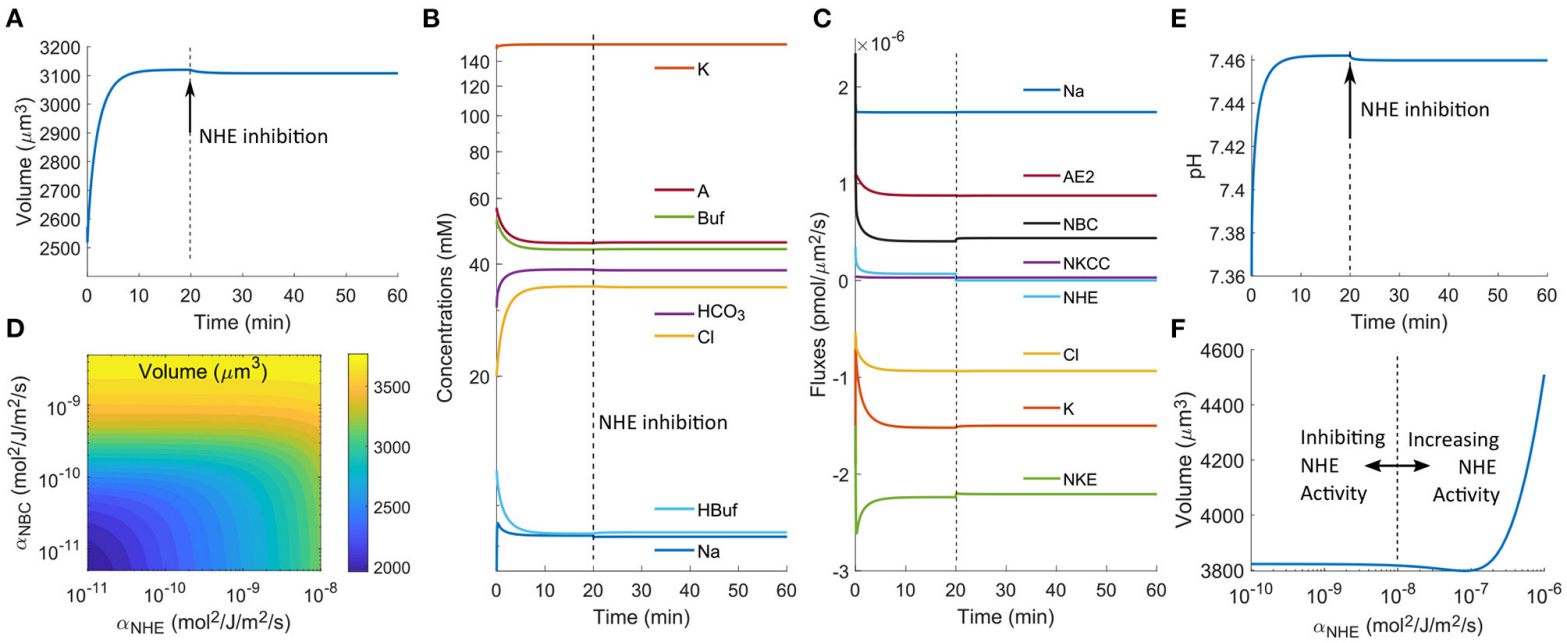
Model predictions of the cellular response upon NHE inhibition at *t*_0_ = 20 min. The permeability coefficient of NBC has been increased by 20 times as compared to the value used in Figures 2, 3. **(A)** Cell volume change upon NHE inhibition. **(B)** Predicted intracellular ion concentrations over time. **(C)** Ion fluxes across various channels and transporters over time. **(D)** Contour plot of the steady-state cell volume as a function of the permeability coefficients of NBC and NHE. **(E)** Changes in the intracellular pH. **(F)** Steady-state cell volume as a function of the permeability coefficient of NHE.

In the above analysis (Figures 2, 3), we have used a relatively small permeability coefficient of NBC, and the model predicts cell volume decrease upon NHE inhibition. If we increase the permeability of NBC by 20 folds (or higher), the cell volume does not decrease (Figure 4A). The intracellular ion concentration of each species also remains almost constant upon NHE inhibition (Figure 4B). This is because when the NBC activities increase, it takes place the role of NHE to import Na^+^, and NHE becomes almost quiescent (compare Figures 3B and 4C). In this case, inhibiting NHE has a minimal effect on the cell volume and intracellular ionic dynamics.

The dependence of cell volume on NBC upon NHE inhibition is a continuous function. Figure 4D shows the volume contour as a function of the permeability coefficients of NBC and NHE. When the NBC expression level is low or none, the cell volume is sensitive to NHE inhibition; when the NBC expression level is high or non-trivial, the cell volume is not affected by NHE inhibition. The dependence of the intracellular pH on NBC is the same as that of cell volume when NHE is inhibited: when NBC is present, the cytoplasm is not predicted to acidify when NHE is inhibited (Figure 4E). Our model thus predicts that NBC may serve as a buffering mechanism to both cell volume decrease and cell acidification (Boron and Boulpaep, 1989) upon NHE perturbation. Indeed, similar phenomena have been observed in the literature. For example, human neutrophils express NBCe2, an isoform of NBC (Giambelluca et al., 2014). Inhibition of NHE on suspended human neutrophils has not been found to change cell volume (Grinstein et al., 1986; Rosengren et al., 1994); inhibition of both NHE and NBC has been found to acidify the cells (Giambelluca et al., 2014) (corresponds to Figure 2H). These experimental results can be interpreted by the mechanisms revealed in this work.

Interestingly, migrating, not suspended, human neutrophils have been found to reduce cell volume with NHE inhibition (Rosengren et al., 1994). Migrating neutrophils have larger volumes than suspended neutrophils (Rosengren et al., 1994). On the other hand, migrating cells exhibit a polarized distribution of NHE (Stroka et al., 2014; Li et al., 2020). We thus speculate that migrating cells have a higher expression level of NHE than suspended cells. Our model does predict that when NHE expression increases, the cell volume increases (Figure 4F). In this case, inhibition of NHE is predicted to reduce cell volume because the contribution of NHE takes over that of NBC. This phenomenon has been observed in an experiment where migrating neutrophils reduces cell volume upon NHE inhibition (Rosengren et al., 1994).

### 3.4. Regulatory Volume Increase by NHE

**Figure 5:**
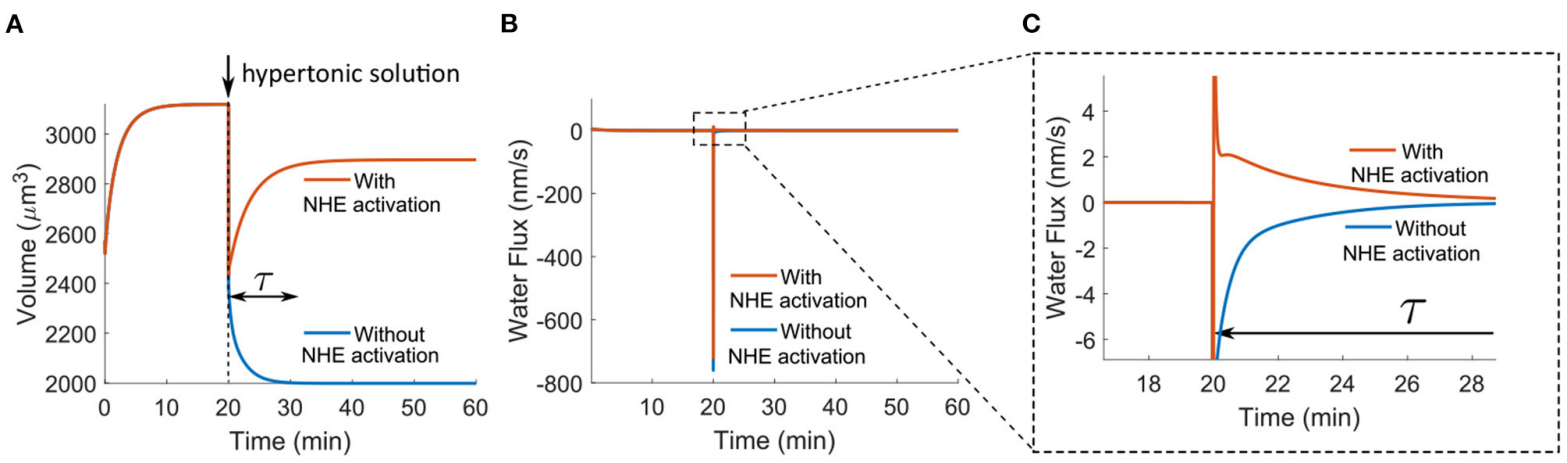
Model predictions of the cell response in the hypertonic solution applied at *t*_0_ = 20 min. The hypertonic solution is applied by adding 50 mM NaCl to the isotonic solution. The permeability coefficient of NBC has been increased by 20 times as compared to the value used in Figures 2, 3. **(A)** Time-dependent response of the cell volume. NHE activation is achieved by increasing the permeability coefficient of NHE by three orders of magnitude at *t*_0_ = 20 min. **(B)** Predicted water flux over time. **(C)** Zoomed-in profile in B.

Here we will briefly discuss the model predictions for one of the most recognized functions of NHE. When cells are transferred from isotonic to hypertonic solutions, cells can (partially) recover their volume, known as regulatory volume increase, and NHE is responsible for this volume recovery (Cala, 1980; Ericson and Spring, 1982; Grinstein et al., 1986; Maeno et al., 2006; Hughes et al., 2010). In the model, we prepare a hypertonic solution by adding 50 mM NaCl to the original extracellular solution. Adding NaCl has been one of the most used methods in the studies of regulatory volume increase (Cala, 1980; Grinstein et al., 1986; Maeno et al., 2006). The model predicts that if the NHE activity remains constant in the entire process, regulatory volume increase does not occur; instead, the cell volume continuously decreases over the transient time, τ (Figure 5A). The regulatory volume increase only happens when NHE is activated upon the initial cell shrink (Figure 5A). In the model, NHE activation is achieved by increasing the permeability coefficient of NHE by three orders of magnitude at the same time as applying the hypertonic solution. Cell shrinkage has been considered to be responsible for this NHE activation; however, the mechanism for this activation remains unclear (Alexander and Grinstein, 2006). In this work, we do not aim to identify the mechanisms for NHE activation; instead, we predict that if this activation occurs, perhaps through other cell signals, regulatory cell volume increase by NHE is observed.

Unlike the temporal volume decrease profile under NHE inhibition (Figure 2A), the volume decrease under hypertonic solution is immediate (*t* ≪ τ) instead of over the transient time τ (Figure 5A). This is because a hypertonic shock leads to an immediate large osmolarity difference and thus an immediate large water outflux (*J*_water_ < 0) two orders of magnitude higher than the flux under NHE inhibition (compare Figures 2B and 5B). On the contrary, the osmolarity difference under NHE inhibition is small (Figure 2E), and the water flux gradually accumulates over the transient time (Figure 2B). When NHE is activated, the large water outflux quickly reverses, and an influx (*J*_water_ > 0) with a small magnitude is predicted (Figure 5C). This influx spans over the transient period of time and the integration of this influx, $\int_{t_{0}}^{t_{0}+\tau }J_{\text{water}}dt$, determines the amount of volume recovery. In our model, the volume recovery time scale, τ, is about 10 min; this time scale is determined by the permeability coefficients of ion channels, transporters, and pumps used in the model. Fast volume recovery on the order of minutes has been observed from experiments on *Amphiuma* red blood cells (Ericson and Spring, 1982), human neutrophils (Grinstein et al., 1986), and mouse choroid plexus epithelial cells (Hughes et al., 2010), while slow recovery on the order of 1 h also exists in *Necturus* gallbladder (Cala, 1980) and HeLa cells (Maeno et al., 2006). Since different cell types express different levels of ion channels, transporters, and pumps, we attribute this time difference to the cell type-specific membrane channel expressions.

### 3.5. Importance of Bicarbonate Export Under AE2 Inhibition

Equation 2 suggests that ${\text{HCO}}_{3}^{-}$ and pH are closely related, and thus ${\text{HCO}}_{3}^{-}$ is also an important ion species to consider whenever H^+^ changes. Given the interconnection between NHE and AE2, here we discuss the cell response under AE2 inhibition. Under physiological conditions, AE2 exports one ${\text{HCO}}_{3}^{-}$ and intakes one Cl^−^ at a time. In this model, NBC is a second transporter that transports ${\text{HCO}}_{3}^{-}$, and the direction of transport depends on the combined electrochemical potential of ${\text{HCO}}_{3}^{-}$ and Na^+^ (Equation 14).

**Figure 6:**
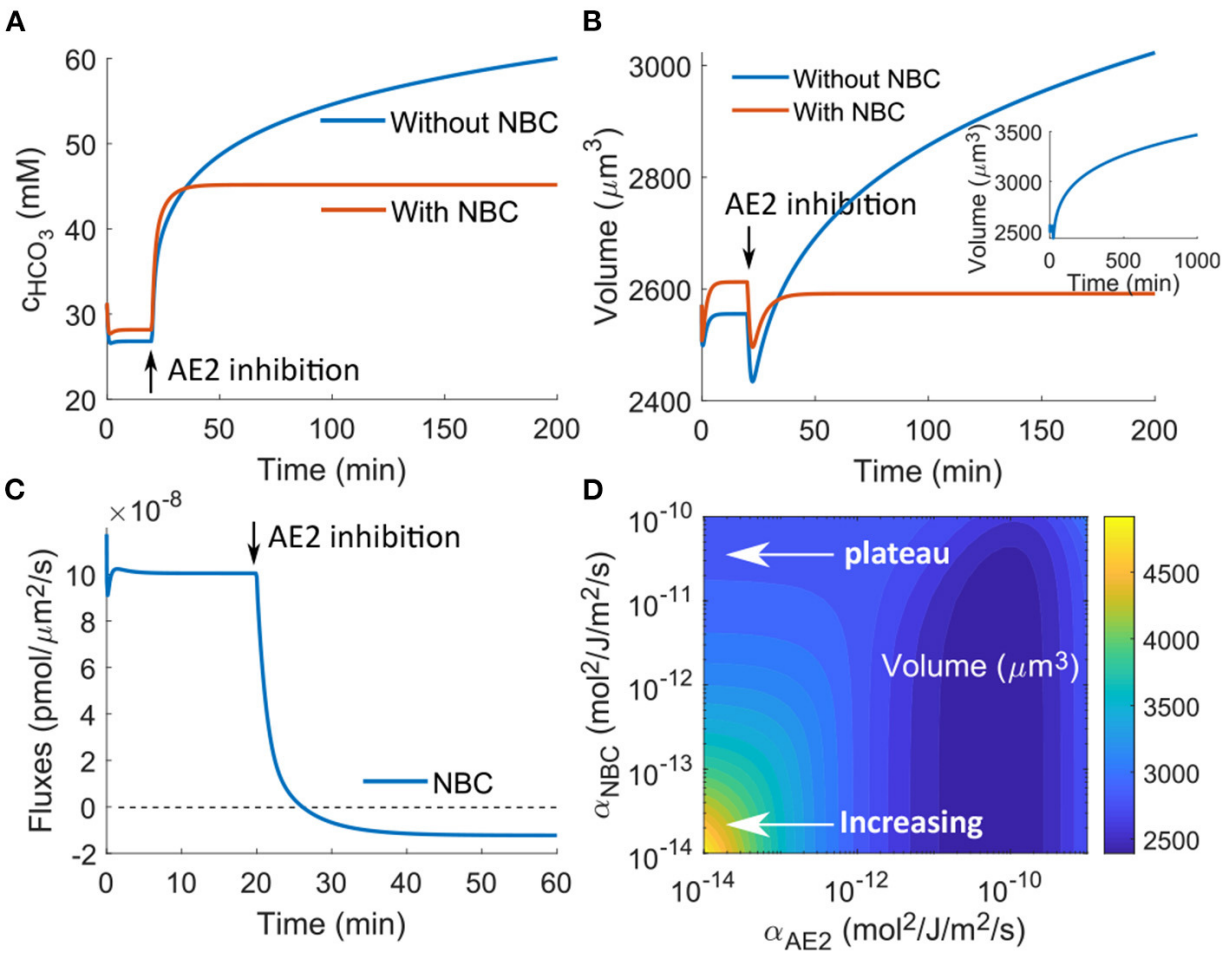
Model predictions of the cellular response upon AE2 inhibition applied at *t*_0_ = 20 min by reducing α_AE2_ by three orders of magnitude. **(A)** Intracellular ${\text{HCO}}_{3}^{-}$ concentration change with and without NBC. **(B)** Model prediction of the cell volume with and without NBC. The insert is a longer time response without NBE, which shows continuous increasing volume in time. **(C)** NBC flux upon AE2 inhibition. **(D)** Contour plot of the cell volume as a function of the permeability coefficients of NBC and AE2.

If in the model we only have AE2 but do not include NBC, then from Equation (9) we can see that AE2 is the only transporter that can export ${\text{HCO}}_{3}^{-}$. When AE2 is inhibited, ${\text{HCO}}_{3}^{-}$ is predicted to accumulate in the cytoplasm, and its concentration increases with time without reaching a steady state even at several hours after inhibition (Figure 6A), which leads to continuous volume increase (Figure 6B). This volume increase is significant, and cell lysis can happen if the degree of AE2 inhibition further increases. However, to the best of our knowledge, AE2 inhibition-induced cell swelling and lysis have not been experimentally observed. On the other hand, continuously increasing intracellular ${\text{HCO}}_{3}^{-}$ will significantly alkalinize the cell, which is also not physiologically favorable. Together, these suggest that cells have additional mechanisms to regulate ${\text{HCO}}_{3}^{-}$ to prevent its excessive accumulation.

NBC is one of the transporters that can prevent the excessive accumulation of ${\text{HCO}}_{3}^{-}$. In the presence of NBC, the concentration of ${\text{HCO}}_{3}^{-}$ and the cell volume reach steady states around 10 min after the perturbation (Figures 6A,B). This ${\text{HCO}}_{3}^{-}$ control comes from the export of ${\text{HCO}}_{3}^{-}$ through the transporter. Before perturbation, the combined electrochemical potential of Na^+^ and ${\text{HCO}}_{3}^{-}$ results in an influx of the two ion species (Figure 6C). As ${\text{HCO}}_{3}^{-}$ accumulates upon AE2 inhibition, the sign of the electrochemical potential reverses, and the NBC begins to export Na^+^ and ${\text{HCO}}_{3}^{-}$. Although the magnitude of the efflux is one order lower than that of the influx before perturbation, when the efflux is integrated over time, it provides sufficient ${\text{HCO}}_{3}^{-}$ removal. For example, if the efflux is on the order of 10^−8^ pmol/μm^2^/s, as predicted by the model (Figure 6C), the amount of ion removal will be on the order of 5 × 10^−3^ pmol over 10 min for a cell of radius *r* = 8 μm. This amount corresponds to ~2 mM change in concentration. Therefore, every 10 min, the efflux can remove an equivalent (2*n*_NBC_) mM concentration of ${\text{HCO}}_{3}^{-}$ from the cell, which is sufficient to control the intracellular concentration.

*In vivo*, the flux through NBC and to what extend this transport can control cell volume depend on the expression level of NBC as well as the degree of AE2 inhibition. We use a contour plot of the steady-state cell volume to show its dependence on the rate constants of NBC and AE2 (Figure 6D). We can see that for small α_NBC_, equivalent to NBC not being present, the cell volume increases as AE2 is progressively inhibited. The cell volume almost doubles at the small end of α_AE2_. For large α_NBC_, equivalent to NBC being present, the cell volume reaches a plateau as α_AE2_ decreases. In between there exists an improved cell volume control upon AE2 inhibition as α_NBC_ increases. The contour plot also suggests that the cell volume will, depending on the expression level of NBC, increase if the AE2 activity increases. The model predicts that the cell volume is a non-monotonic function of the AE2 activity if NBC is absent and approaches a constant if NBC is present. The exact expressions of AE2 and NBC are likely to be cell-type dependent, and it thus can happen that different cells will respond differently upon perturbation and will have different concentrations of intracellular ${\text{HCO}}_{3}^{-}$. On the other hand, if mammalian cells are programmed to maintain constant volume upon reasonable perturbation, the expression level of NBC should be high in all cells. It can be interesting to see from experiments how cell volume modulates when AE2 is inhibited or activated, for both control and NBC-inhibited cells.

### 3.6. Implication of NBC on Cell Volume Regulation Upon NKE Inhibition

In the previous example, AE2 and NBC are the only two transporters that transport ${\text{HCO}}_{3}^{-}$; therefore, it is relatively easy to provide a physical interpretation of the dynamics of ${\text{HCO}}_{3}^{-}$ upon AE2 inhibition. When it comes to ions that are transported by multiple channels, transporters, and pumps, such as Na^+^, the results may not seem to be straightforward. In this section, we will discuss how NBC and ${\text{HCO}}_{3}^{-}$ are involved in the volume regulation when NKE is inhibited.

**Figure 7:**
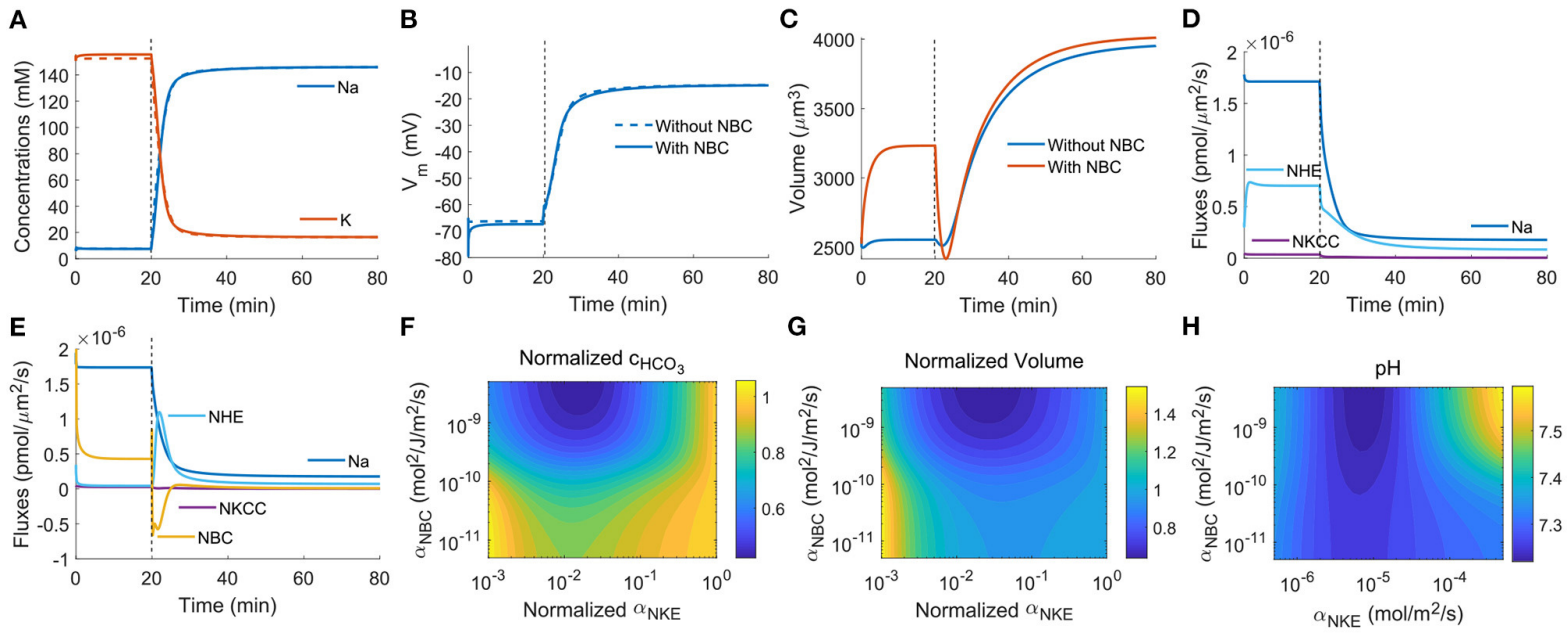
Model predictions of the cellular response upon NKE inhibition applied at *t*_0_ = 20 min (indicated by dashed vertical lines) by reducing α_NKE_ by three orders of magnitude. **(A)** Intracellular Na^+^ and K^+^ concentrations. Dashed lines: without NBC. Solid lines: with NBC. **(B)** Cell membrane potentials. **(C)** Cell volumes. **(D)** Fluxes across the channels, transporters, and pumps that transport Na^+^. NBC is absent. **(E)** Fluxes across the channels, transporters, and pumps that transport Na^+^. NBC is present. **(F)** Contour plot of the steady-state, normalized intracellular ${\text{HCO}}_{3}^{-}$ concentration as a function of the permeability coefficient of NBC and the normalized permeability coefficient of NKE. α_NKE_ indicates the degree of NKE activity. The concentration of ${\text{HCO}}_{3}^{-}$ is normalized with respect to the case without NKE inhibition so that the vertical line on the right end of the contour has value 1. **(G)** Contour plot of the steady-state, normalized cell volume as a function of the permeability coefficient of NBC and the normalized permeability coefficient of NKE. **(H)** Contour plot of the steady-state intracellular pH as a function of the permeability coefficients of NBC and NKE.

As an active pump that transports Na^+^ and K^+^ against their electrochemical potential, NKE is known to regulate the intracellular Na^+^ and K^+^ concentrations and the membrane potential (Gadsby, 2009). When NKE is inhibited, Na^+^ accumulates in the cytoplasm, and the K^+^ concentration starts to reduce, resulting in almost a flip of cytoplasmic concentrations of Na^+^ and K^+^ compared to the concentrations in normal conditions (Figure 7A). Meanwhile, the cell membrane is depolarized to ~20 mV (Figure 7B). We do not see a quantitative difference in Na^+^ and K^+^ concentrations and the membrane potential with and without NBC (Figures 7A,B); however, NBC helps to stabilize the cell volume to some extend when NKE is inhibited (Figure 7C). The cell is predicted to experience about a 50% volume increase when NBC is absent. But the increase is only about 20% when NBC is present. Of note, the presence of NBC also increases the baseline cell volume due to the additional ions brought by the transporter. We speculate that in wet-lab experiments, 50% volume increase is not expected because although too much ouabain, an NKE inhibitor, will kill cells, a small amount of ouabain (e.g., up to 50 μM for MDA-MB-231) alone has not been found to cause cell lysis (Gould III et al., 2018; Maity et al., 2020). We will next analyze the role of NBC in this process.

When NBC is absent, passive Na^+^ channel, NHE, and NKCC are the three channels/transporters that transport Na^+^ in addition to the NKE that is inhibited. The direction of the fluxes through the three channels/transporters is determined by the gradient of the electrochemical potentials of the relevant ion species. Therefore, each of the three channels/transporters has the possibility to reverse its direction of transport. Although the intracellular Na^+^ concentration significantly increases upon NKE inhibition, it is not large enough to reverse the direction of the passive Na^+^ channel, NHE, or NKCC (Figure 7D). When NBC is present, the fluxes through the passive Na^+^ channel and NKCC do not change compared to the case without NBC, but the flux through NHE is different (Figure 7E). We first notice that the direction of flux through NBC is reversed right after NKE inhibition. This is due to the sudden increase of the intracellular Na^+^ concentration (Figure 7A), which leads to a reversed gradient of the combined potential of Na^+^ and ${\text{HCO}}_{3}^{-}$ across the cell membrane. The reversal of NBC leads to an efflux of ${\text{HCO}}_{3}^{-}$, with a factor of *n*_NBC_ more than the flux of Na^+^. This ${\text{HCO}}_{3}^{-}$ efflux acidifies the cell, which activates NHE, as seen by a large peak of the NHE flux following NHE inhibition (Figure 7E). The activation of NHE also brings in Na^+^, which explains why the concentration of Na^+^ is unchanged compared to the case with NBC (Figure 7A) even if NBC can export Na^+^.

The model thus predicts that the presence of NBC has a significant effect on the cytoplasmic ${\text{HCO}}_{3}^{-}$. Figure 7F is a contour of the steady-state, normalized intracellular ${\text{HCO}}_{3}^{-}$ concentration as a function of the permeability coefficient of NBC and the normalized permeability coefficient of NKE. The normalized α_NKE_ indicates the degree of NKE inhibition; the smaller it is, the more inhibited the pump is. The concentration of ${\text{HCO}}_{3}^{-}$ is normalized with respect to the case without NKE inhibition so that the normalized concentration on the vertical line on the right end of the contour has value 1. When α_NBC_ is large (on the same order of α_0_), corresponds NBC being present, the concentration of ${\text{HCO}}_{3}^{-}$ decreases with NKE inhibition, same as the analysis from Figure 7E. This concentration decrease helps to prevent significant cell volume increase (Figure 7C). When α_NBC_ is small (≪α_0_), corresponds NBC being absent, the concentration of ${\text{HCO}}_{3}^{-}$ initially decreases and then increases back to the original value as NKE is further inhibited. Here we plot the normalized volume with respect to the volume without NKE inhibition to show the relative volume change. The original concentration of ${\text{HCO}}_{3}^{-}$ without inhibition in the presence of the NBC is higher than that without NBC.

The normalized cell volume follows the same pattern as the normalized intracellular ${\text{HCO}}_{3}^{-}$ concentration (compare Figures 7G,F). The original cell volume is also larger when NBC is present compared to that when NBC is absent. This result shows that the size homeostasis of the cell depends on the expression levels of ion channels and transporters. Our work thus suggests that the volume difference across different cell types maybe is the result of their expression levels of ion transporters, in addition to possible differences in protein content.

The model also predicts that with slight NKE inhibition (a 10^−1^ or 10^−2^ change in expression), the cell volume will decrease instead of increasing (compare Figure 7G). This result can be model-dependent as it is possible that including additional channels, transporters, and pumps may change the results. But on the other hand, it is also possible that NKE has a duel effect on cell volume depending on its activities. Of note, when NKE permeability is further reduced beyond the range plotted in Figure 7G, the cell volume will continue to increase, implying that excessive inhibition of NKE eventually causes cell lysis, which is consistent with the fact that complete inhibition of NKE leads to cell death (Pchejetski et al., 2003; Valente et al., 2003; Chou et al., 2018).

Although NKE itself does not involve H^+^ or ${\text{HCO}}_{3}^{-}$, its inhibition has an impact on the intracellular pH through NHE (Gatti and Christen, 1985). Our model predicts the same phenomenon. Since the presence of NBC tends to reduce the intracellular ${\text{HCO}}_{3}^{-}$ concentration upon NKE inhibition, it also acidifies the cell as a result (compare Figure 7H). The model thus provides generic mechanisms that describe the dynamics among different ion species and their implications on both cell volume and pH.

### 3.7. Predictions of Cell Type-Dependent Cell Properties

**Figure 8:**
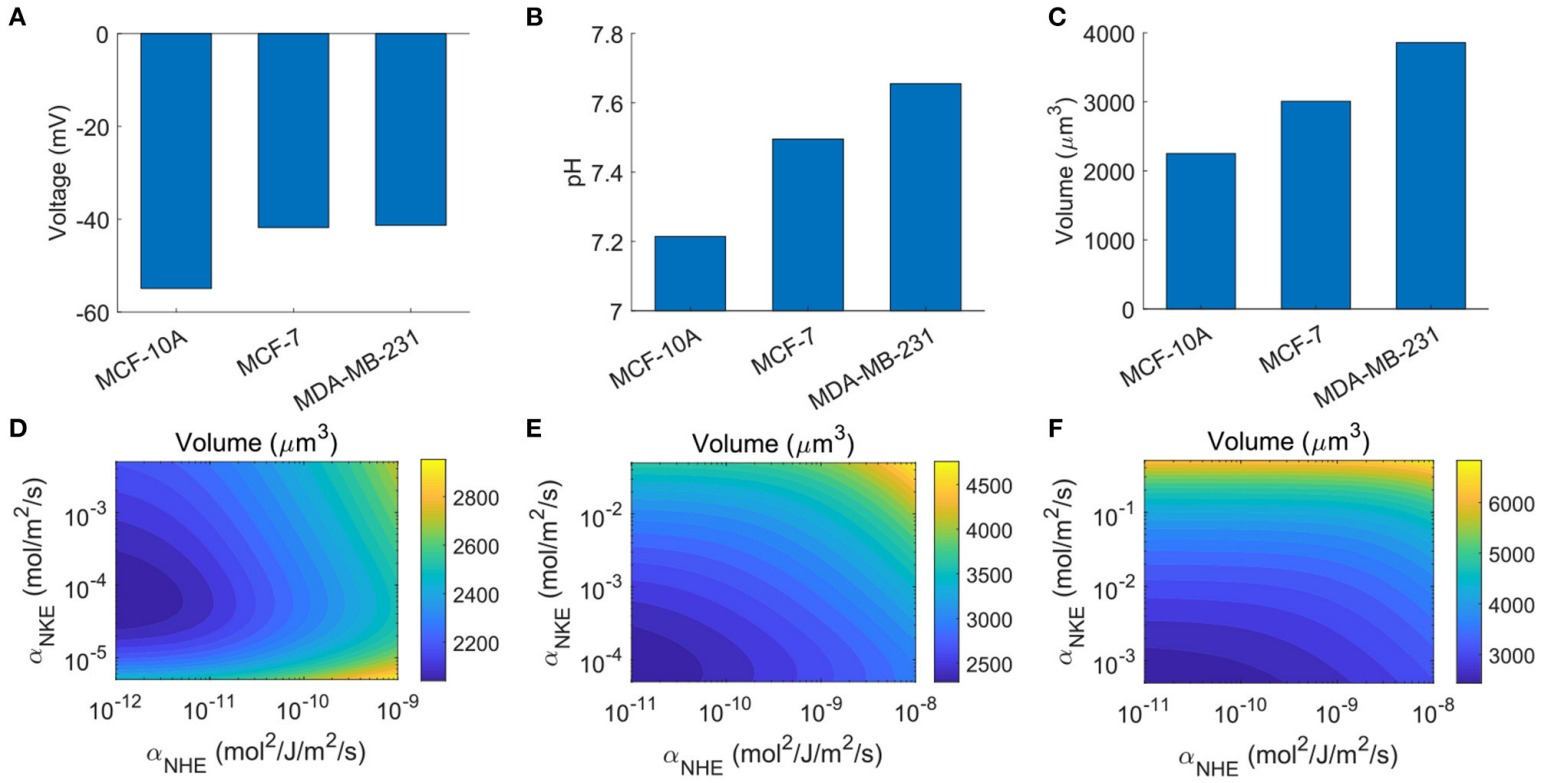
Examples of cell type-dependent cell properties. **(A)** Computed cell membrane potentials. **(B)** Model prediction on the intracellular pHs. **(C)** Model prediction on the cell volume of the three cell types, assuming the cell protein contents are identical. **(D–F)** The contour of cell volumes for MCF-10A, MCF-7, and MDA-MB-23, respectively, as functions of NKE and NHE activities. The variation of NKE and NHE activities ranges from 10^−2^*to*10 folds based on the values listed in Table 2.

In this last section, we provide an example of how different cell types can respond differently upon perturbations of ion channels, transporters, and pumps. The following three common breast cell types will be studied: MCF-10A, normal breast cell line; MCF-7, weakly malignant breast cancer cell line; MDA-MB-231, highly malignant, metastatic breast cancer cell line. These three breast cell lines are known to have varying expression levels of different ion channels, transporters, and pumps, including NHE (Amith and Fliegel, 2013; Boedtkjer et al., 2013), NBC (Boedtkjer et al., 2013; Alka and Casey, 2014), AE2 (Hwang et al., 2019), and NKE (Khajah et al., 2018). These variations provide the qualitative basis for the cell type-dependent parameters used in the model (Table 2). We also adjust the permeation coefficient of the passive sodium channel so that the predicted membrane potential of the three cell types (Figure 8A) matches with experimental measurement (Marino et al., 1994; Yu et al., 2017). Since our model considers a subset of ion channels, transporters, and pumps that are present in cells, parameters used here for the three cell types are not meant to be exact, but rather a qualitative representation of different cell properties.

**Table 2:** Cell type-specific parameters.

**Parameter**	**MCF-10A**	**MCF-7**	**MDA-MB-231**	**Source**
α_Na, *p*_ (mol^2^/J/m^2^/s)	0.2α_0_	0.4α_0_	0.55α_0_	Fitted (see text)
α_NKE_ (mol/m^2^/s)	$1\times 10^{4}\alpha_{0}/RT$	$1\times 10^{6}\alpha_{0}/RT$	$1\times 10^{7}\alpha_{0}/RT$	Based on (Khajah et al., 2018)
α_NHE_ (mol^2^/J/m^2^/s)	$1\times 10^{-2}\alpha_{0}$	$1\times 10^{-1}\alpha_{0}$	$1\times 10^{-1}\alpha_{0}$	Based on (Boedtkjer et al., 2013)
α_AE2_ (mol^2^/J/m^2^/s)	$1\times 10^{-2}\alpha_{0}$	$9\times 10^{-3}\alpha_{0}$	$8\times 10^{-3}\alpha_{0}$	Based on (Hwang et al., 2019)
α_NBC_ (mol^2^/J/m^2^/s)	$1\times 10^{-3}\alpha_{0}$	$2\times 10^{-3}\alpha_{0}$	$3\times 10^{-3}\alpha_{0}$	Based on (Boedtkjer et al., 2013)

*The parameters follow the qualitative trends of the intensities from either Western Blot or immunofluorescence discovered in the experiments. The rest of the parameters are identical to those listed in Table 1*.

With the above setup, the model predicts higher intracellular pH in breast cancer cells than that in the normal breast cell (Figure 8B). This prediction is consistent with the fact that there is an alkalinization of the cytoplasm in breast cancer cells due to the elevated activities of NHE1 (Amith and Fliegel, 2013) and NBCn1 (Alka and Casey, 2014). The model also predicts that the rest cell volume increases progressively with the malignancy level (Figure 8C). The trend of cell volume is consistent with the trend of pH. This is because, as discussed in the above sections, the concentration of bicarbonate increases with increasing pH, which leads to increased cell volume. We note that the cell protein content, or *N*_*A*_, is assumed to be the same. In reality, the cell protein content could be different, but there is currently no data on this.

Based on the analysis so far, it is expected that the three cells will show different cell volume variation patterns when their ion channels, transporters, or pumps are perturbed. Indeed, if we allow NKE and NHE expression levels to vary based on the values listed in Table 2, the three cells exhibits different cell volume changes (Figure 8D, MCF-10A; Figure 8E, MCF-7; and Figure 8F, MDA-MB-231). The model predicts that all three cell types respond to NKE and NHE. The volume of MDA-MB-231 is more sensitive to NKE while the volume of MCF-10A is more sensitive to NHE. These predictions may be different if more ion channels, transporters, and pumps are included in the model. A comprehensive study of cell type-dependent cell properties requires more data on the cell channel composition.

MCF-7 and MDA-MB-231 spheroids indeed have reduced volume when NHE is inhibited (Chang et al., 2014; Rolver et al., 2020), and MCF-7 seems to be more sensitive than MDA-MD-231 on NHE inhibition (Rolver et al., 2020), which is consistent with our model prediction (compare Figures 8E,F). But this tumor volume reduction was probably due to downstream effects of NHE inhibition instead of a direct ion flux change across the cell membrane. We thus leave it as an open question that how expression levels of ion channels, transporters, and pumps affect cell volume *in vivo*.

### 3.8. A Few Notes on the Parameters and Alternative Models

**Figure 9:**
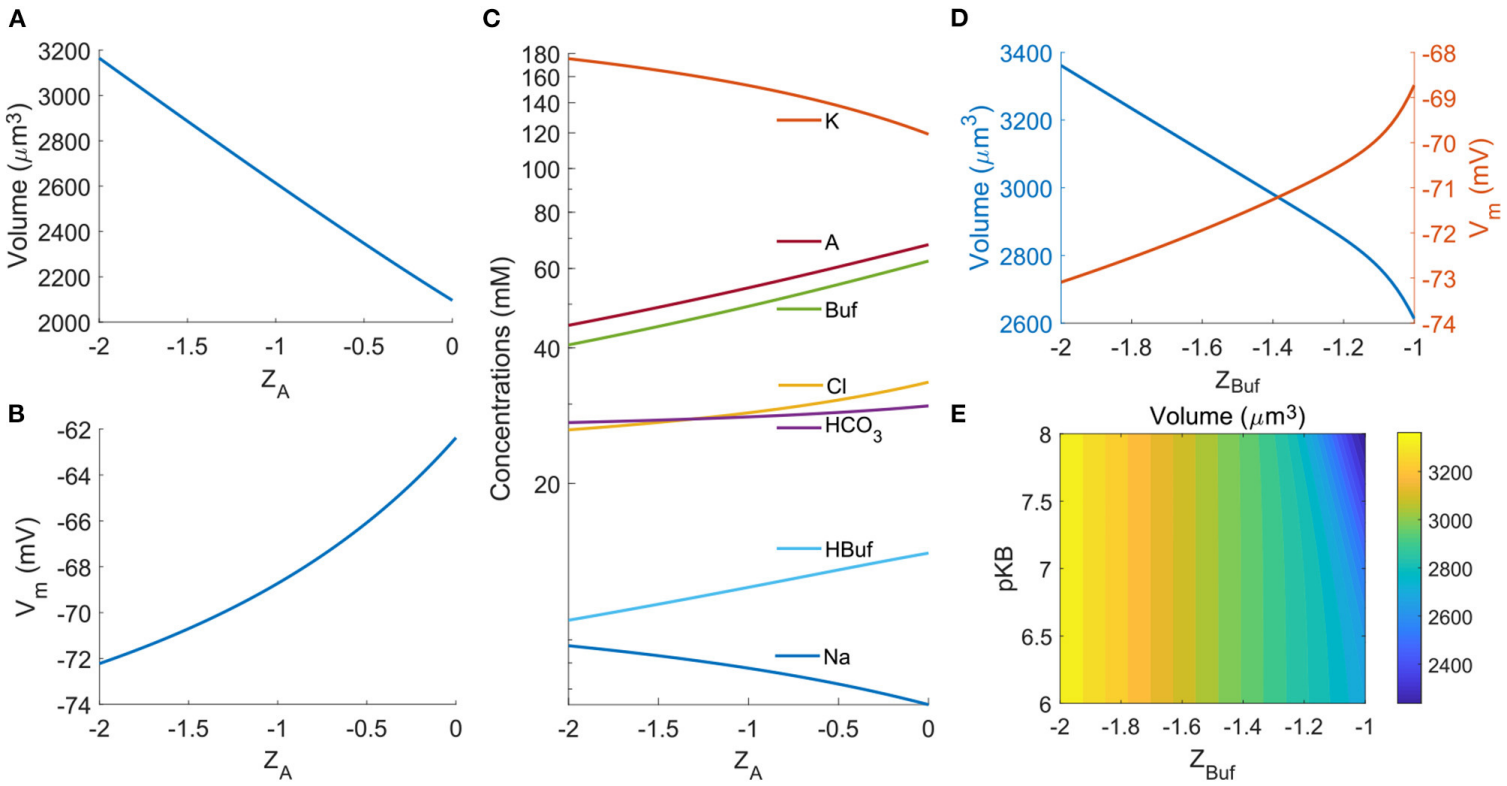
Impact of *Z*_*A*_ and *Z*_Buf_. **(A)** Cell volume as a function of *Z*_*A*_. **(B)** Membrane potential as a function of *Z*_*A*_. **(C)** Intracellular ion concentrations as function of *Z*_*A*_. **(D)** Cell volume and Membrane potential as functions of *Z*_Buf_. **(E)** Contour of the cell volume as a function of the effective p*K*_*B*_ and *Z*_*A*_.

We begin with studying the impact of the effective valences of some of the species. The average charge of the non-permeable organic molecules and proteins, i.e., the valence *Z*_*A*_, is a potential variable that can vary across cell types. The model predicts that the cell volume increases with the magnitude of the negative *Z*_*A*_ (Figure 9A). The cell also becomes further polarized by several mV per unit *Z*_*A*_ change as the proteins carry more negative charges (Figure 9B). The concentrations of intracellular ions will adjust correspondingly. As expected, in general, the negatively charged ions decrease their concentrations with the magnitude of the negative *Z*_*A*_ while positively charged ions go in the opposite direction (Figure 9C). The trend of the dependence of various cell responses on *Z*_*A*_ is consistent with the model prediction by Kay (2017).

Different buffer solutions can give rise to different valences of the unprotonated buffers. In the model, we can vary the effective lumped valence of all buffer solutions. Similar to the impacts from the effective valence of the organic molecules and proteins, large |*Z*_Buf_| leads to larger cell volume and more polarized cell compared to those from a small |*Z*_Buf_| (Figure 9D). Since different buffer solutions will have different reaction equilibrium constant, here we also investigate the effective p*K*_*B*_ values on the cell volume. The model predicts that p*K*_*B*_ only affects the cell volume when *Z*_Buf_ is close to −1 (Figure 9E).

**Figure 10:**
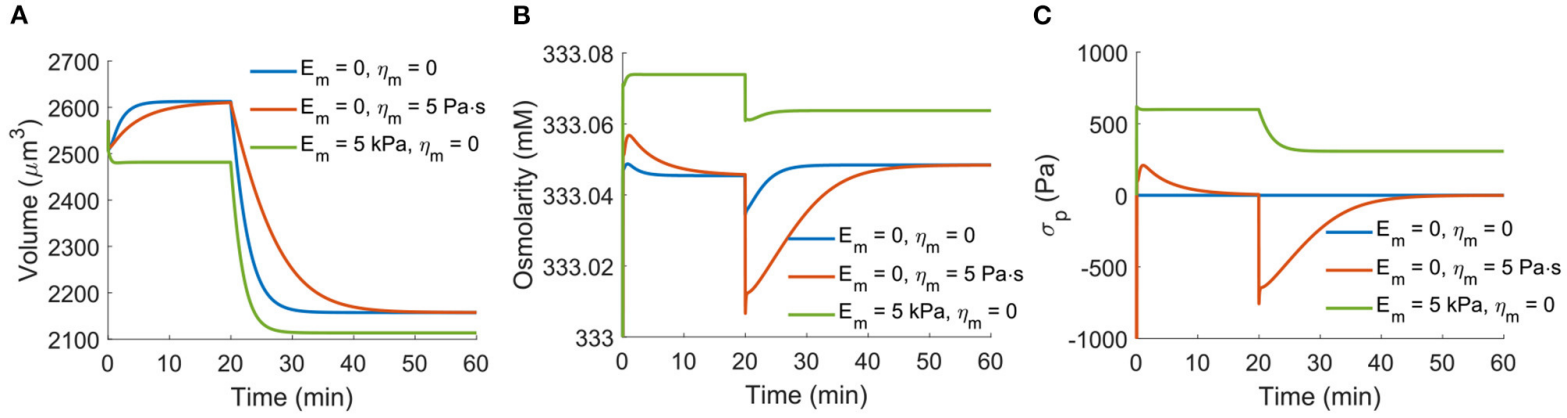
Impact of the cell cortical mechanics on the responses of the cell under NHE inhibition as shown in Figure 2. The data from *E*_*m*_ = η_*m*_ = 0 is the same as the data in Figure 2. The reference cell volume is taken as *r*_0_ = 7.5 μm. **(A)** Cell volume for different cortical properties. **(B)** Total intracellular osmolarity for different cortical properties. **(C)** The passive cortical stress, σ_*p*_, for different cortical properties.

In the model, we have assumed a constant cortical tension dominated by the active myosin contractility. Here we briefly examine a model with a more general cortical mechanics. The total cortical stress is

(38) $$\begin{array}{l} \sigma =\sigma_{p}+\sigma_{a},\;\sigma_{p}=E_{m}\left(\frac{r}{r_{0}}-1\right)+\frac{\eta_{m}}{r_{0}}\cdot \frac{dr}{dt}, \end{array}$$

where *E*_*m*_ is the cortical elasticity, η_*m*_ is the cortical viscosity, and *r*_0_ is a reference cortical radius. We re-examine the case of NHE inhibition as shown in Figure 2. With cortical viscosity, the transient time scale increases (Figure 10A). The presence of the cortical elasticity increases the cortical stress and thus reduces the cell volume (Figure 10A). With the increase of the total cortical stress, the intracellular osmotic pressure also increases (Figure 10B). While the cortical elasticity generates positive tensile stress in the cortex, the viscosity generates negative compression stress as the cell shrinks (Figure 10C). We would like to mention that due to the continuous remodeling of the cortex, in reality, there is no defined reference cell radius *r*_0_, and thus there does not exist cortical elasticity. The passive cortical mechanics will mostly be the viscous part.

We have also assumed non-permeable protonated buffer solutions in the model. Since some uncharged buffer solutions can be permeable to the lipid bilayer, such as acetic acid or lactic acid, here we examine the case with permeable protonated buffer solutions, meaning the HBuf moves freely in and out of the cell. The extracellular space is still considered an infinite reservoir with constant properties. In this regard, we prescribe the concentration of HBuf instead of the total amount of intracellular buffer amount, i.e., $c_{\text{HBuf}}=c_{\text{HBuf}}^{0}=\text{const.}$ is given. The unprotonated buffer concentration is determined by the pH, i.e., $c_{\text{Buf}}=c_{\text{HBuf}}10^{\text{pH}-\text{p}K_{B}}$ and $c_{\text{Buf}}^{0}=c_{\text{HBuf}}^{0}10^{{\text{pH}}_{0}-\text{p}K_{B}}$. The rest of the modeling framework remains the same. We also keep all the parameters the same with the only change of the extracellular $c_{{\text{HCO}}_{3}}^{0}$ from 35 mM (Table 1) to 25 mM. The 10 mM difference goes to $c_{\text{HBuf}}^{0}$. The extracellular space is adjusted to electroneutral through *c*_*A*_ and *c*_G_, and the total extracellular osmolarity is kept at 365 mM.

**Figure 11:**
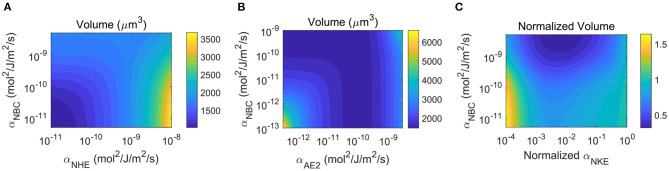
Key model predictions with permeable protonated buffer solution. The predicted trends with permeable protonated buffer solution are the same as a model with non-permeable protonated buffer solution. **(A)** Contour plot of the cell volume as a function of the permeability coefficient of NBC and NHE. This plot is a counterpart of Figure 4D. **(B)** Contour plot of the cell volume as a function of the permeability coefficient of NBC and AE2. This plot is a counterpart of Figure 6D. **(C)** Contour plot of the normalized cell volume as a function of the permeability coefficient of NBC and the normalized permeability coefficient of NKE. This plot is a counterpart of Figure 7G.

We select the three most informative results and compare these to the original model. Figures 11A–C correspond to Figures 4D, 6D, 7G respectively. Although there is some quantitative difference between the two model choices, the overall trends remain the same. All these results show that the presence of NBC helps to stabilize the cell volume when the cell is under channel/transporter/pump perturbation. Therefore, the conclusion of our model prediction is robust.

## 4. Discussion and Conclusions

In this work, we performed computational experiments based on a mathematical model to analyze how hydrogen and bicarbonate are involved in cell volume regulation. We included several commonly expressed ion transporters/pumps as well as passive ion channels and focused on generic model predictions on the cell volume and cell osmolyte concentration. For example, at a steady state, the cell volume is tightly correlated with the cell osmolarity, and changes in cell volume result in tiny ion concentration changes (much <1 mM). Therefore, the total intracellular solute concentration remains almost constant regardless of the cell volume. This is consistent with our previous results on cell volume variations as a function of membrane potential (Yellin et al., 2018). In this work, we have focused on a spherical cell. One of the features of a spherical cell is its non-polarity. The trends of volume change predicted by this model still apply to non-polarized cells even if they do not adopt a spherical shape. For adhered cells, the force-balance relationship and the geometry of the cell will be different, but this should only change the results qualitatively. For polarized cells, such as cell migration in confined channels, our model provides information on localized water influx and efflux depending on the membrane protein expression at different spatial locations of the cell membrane.

The model involves a number of parameters (Table 1). Some parameters describe the intrinsic molecular properties of ion channels, transporters, and pumps (e.g., dependence on concentration differences, cortical/membrane tension, pH, and voltage). Other parameters, such as the permeability coefficients of channels, transporters, and pumps (α's), describe the state of ion channel activation as well as the overall expression level and therefore depend on the cell type. We find that the predicted cell response is most sensitive to the permeability coefficients: α's. We noted that these permeability coefficients probably depend strongly on cell signaling and regulatory mechanisms, e.g., post-translational modification and Ca^2+^ binding. α_0_ sets the overall cell response time scale to external perturbations. One order of magnitude change in α_0_ leads to about one order of magnitude change of the transient response time. The activity of NKE plays a major role in controlling the intracellular Na^+^ concentration and membrane potential. One order of magnitude decrease in α_NKE_ leads to roughly 40 mM increase in intracellular Na^+^ concentration and 20 mV depolarization. Activities of AE2 and NKE have non-monotonic effects on the cell volume (Figures 6D, 7G) while the effects from NHE and NBC are monotonic (Figures 3F, 6D, 7G). The sensitivity of cell volume to these four permeability coefficients of AE2, NKE, NHE, and NBC depends on their relative ratios. For example, from Figure 6D, we can see that the cell has a larger volume change with the inhibition of AE2 when NBC is absent and vice versa. This is because in this model AE2 and NBC are the only two transporters that can remove ${\text{HCO}}_{3}^{-}$ from the cell. If one of the transporters is absent, the cell lacks redundant mechanisms for maintaining ${\text{HCO}}_{3}^{-}$ homeostasis; inhibition of the remaining transporter thus leads to significant cell volume change.

Given the large number of channels and transporters in mammalian cells and significant variations in the expression profiles from cell type to cell type, we do not claim that our work represents a minimal model nor a sufficient model. We aim to develop a model that captures the typical trends of the cellular response under the influence of hydrogen and bicarbonate. Some small variations of the model are also possible. For example, in the model, we have included AE2, which is pH-gated. The two other isoforms of AE2, namely AE1 and AE3, are pH-independent (Sterling and Casey, 1999). Non-pH-gated AEs can be lumped into the model by varying the pH gating parameters if needed. A truly comprehensive model requires concerned effort to generate a consistent set of data. The existence of redundant mechanisms also increases the complexity of a comprehensive model. For example, NBC can be replaced by a combination of other transporters that can remove Na^+^ and ${\text{HCO}}_{3}^{-}$ when these two ion species start to accumulate in the cytoplasm. The choice of what and how many ion transporters to include depends on the physical problems to be addressed. In a problem involving pH, the inclusion of NHE, AE2, and NBC is necessary. However, in problems where the variation of pH and ${\text{HCO}}_{3}^{-}$ is not crucial, these transporters may be left out.

In this model, we did not include Ca^2+^, which is also an important ion in the cytoplasm. Some channels transport Ca^2+^ and Na^+^ and can take the place of NBC if the Na^+^ dynamics is considered. In addition to channels that directly transport Ca^2+^, Ca^2+^ is a second messenger that often regulates the activity of various ion channels and transporters (Mahnensmith and Aronson, 1985; Garty and Palmer, 1997; Ruiz et al., 1999; Shieh et al., 2000). For example, the activity of NHE1 can be modulated by Ca^2+^ through TRPV4, determining pH-dependent actin polymerization, providing mechanical stability to delineate lamellipodia structure, and defining the efficiency of cell migration (Di Giusto et al., 2020). Ca^2+^ also affects the structure and dynamics of the cytoskeleton (Oertner and Matus, 2005; Hepler, 2016; Zhao et al., 2019; Maity et al., 2020), which further impacts the distribution and function of ion channel (Levina et al., 1994; Steele and Fedida, 2014; Vallés et al., 2015). The dependence of ion channel activity on Ca^2+^ and other biochemical signals provides additional regulatory mechanisms for cells to maintain ion homeostasis under environmental perturbation. For example, the contour plot in Figure 7G suggests that the cell volume is a non-monotonic function of the NKE activity for fixed NBC activity. If cells have active control mechanisms that vary NBC activity, cells can also follow a constant volume contour when NHE activity decreases through reducing NBC activity followed by increasing NBC activity. Such active mechanisms remain to be studied.

The active stress generated by cytoskeleton contraction, σ_*a*_, plays an important role by setting the hydraulic pressure in the cytoplasm. The ionic fluxes in Equations 24–27 are all functions of ion concentrations, and therefore at steady state, it is ion channels, transporters, and pumps that set the overall cell solute concentration, and therefore the total osmolarity difference, ΔΠ. The cell volume is then determined by a ΔΠ and Δ*P* through force balance. Note that force balance would change depending on the cell surface curvature and cell geometry (Perez-Gonzalez et al., 2019). Therefore, this model could potentially explain how cells sense their size and geometry in different culture conditions, adhesion, substrate stiffness, and extracellular matrix, provided that we understand how cell active contraction is governed in these conditions. Indeed, it is likely that the cell actively controls σ_*a*_ together with ion fluxes and therefore achieves a desired cell volume. By obtaining activity levels of ion channels/transporters/pumps, cytoskeletal motors, and the cell adhesion geometry, we can directly predict the homeostatic cell volume and ultimately explain why different cell types have different volumes.

In this work, we have shown that the expression levels of ion channels, transporters, and pumps determine cells' ability to regulate their volumes. Cells in pathological conditions may alter the expressions or activities of ion channels and transporters compared to physiological conditions (Amith and Fliegel, 2013; Boedtkjer et al., 2013; Alka and Casey, 2014; Gould III et al., 2018; Khajah et al., 2018; Hwang et al., 2019). The ability to alter ion channel activity and expression allows the cell to adapt to environmental changes. The regulation of ion channel activity may be one of the hallmarks of diseases since diseased cells may develop alternative mechanisms for survival and motility. For example, it was shown that ion fluxes may be used by cancer cells to migrate in confined spaces (Stroka et al., 2014). Moreover, cell ionic content can directly influence protein translation and DNA replication (Li et al., 2020). Therefore, the mechanism of cell ionic homeostasis is of central importance in cell biology, and our model has implications for water- and osmosis-driven cell migration, and other biological problems of interest.

## Data Availability Statement

The original contributions presented in the study are included in the article/supplementary material, further inquiries can be directed to the corresponding author/s.

## Author Contributions

YL and SS developed the model. YL ran the simulations. YL, XZ, and SS wrote the manuscript. All authors contributed to the article and approved the submitted version.

## Conflict of Interest

The authors declare that the research was conducted in the absence of any commercial or financial relationships that could be construed as a potential conflict of interest.
